# Biologically-Based and Physiochemical Life Support and In Situ Resource Utilization for Exploration of the Solar System—Reviewing the Current State and Defining Future Development Needs

**DOI:** 10.3390/life11080844

**Published:** 2021-08-18

**Authors:** Ryan J. Keller, William Porter, Karthik Goli, Reece Rosenthal, Nicole Butler, Jeffrey A. Jones

**Affiliations:** Center for Space Medicine, Baylor College of Medicine, Houston, TX 77030, USA; Will.Porter@bcm.edu (W.P.); Karthik.Goli@bcm.edu (K.G.); Reece.Rosenthal@bcm.edu (R.R.); Nicole.Butler@bcm.edu (N.B.); jajones@bcm.edu (J.A.J.)

**Keywords:** life support, atmospheric revitalization, in situ resource utilization, space exploration, planetary habitat, transfer vehicle, BLSS, ISRU, cyanobacteria, methane

## Abstract

The future of long-duration spaceflight missions will place our vehicles and crew outside of the comfort of low-Earth orbit. Luxuries of quick resupply and frequent crew changes will not be available. Future missions will have to be adapted to low resource environments and be suited to use resources at their destinations to complete the latter parts of the mission. This includes the production of food, oxygen, and return fuel for human flight. In this chapter, we performed a review of the current literature, and offer a vision for the implementation of cyanobacteria-based bio-regenerative life support systems and in situ resource utilization during long duration expeditions, using the Moon and Mars for examples. Much work has been done to understand the nutritional benefits of cyanobacteria and their ability to survive in extreme environments like what is expected on other celestial objects. Fuel production is still in its infancy, but cyanobacterial production of methane is a promising front. In this chapter, we put forth a vision of a three-stage reactor system for regolith processing, nutritional and atmospheric production, and biofuel production as well as diving into what that system will look like during flight and a discussion on containment considerations.

## 1. Introduction

When planning long-distance spaceflight missions, it becomes critical to create ways to reduce IMLEO (initial mass in low earth orbit) while also ensuring that the systems that increase IMLEO are reliable and have enough redundancies to ensure the success of the mission. Multiple probes and rovers have already been sent to Mars, but future missions will add new complications in keeping humans alive and returning them home safely, requiring food, oxygen, carbon dioxide scrubbing, and propellants for the transit vehicle and DAV (Descent/Ascent Vehicle). The current systems utilized on board spacecraft are physical-chemical, relying on both renewable and nonrenewable resources that are limited and require occasional resupply [1]. The current systems will not support a long-duration mission outside of LEO (Low Earth Orbit), and resupply to Mars, for example, will be both long and expensive. BLSS/ISRU (Bioregenerative Life Support System/In-Situ Resource Utilization) attempts to tackle these problems.

Lunar and Martian regolith contain many useful elements and compounds for survival, but at the moment, they do not exist in a form that satisfies our life support needs. The Martian atmosphere contains 95% carbon dioxide and is very thin and inhospitable for humans and broad forms of life. Cyanobacteria are ancient photosynthetic organisms on Earth that are believed to be responsible for terraforming and oxygenating the planet to support higher orders of life. They are very effective sources of oxygen production, produce many useful compounds, and are used throughout the world as a nutritional supplement for their high protein content and wide resumé of vitamins, minerals, and antioxidants. Such a versatile organism has the potential to revolutionize the way we operate life support systems in space. Inflight BLSS would need to provide oxygen and food for the crew for interplanetary travel, thereby reducing dependence on foodstuffs and oxygen stores taken from Earth. For Planetary ISRU, a three-stage system is being proposed. Stage 1 will be responsible for bioweathering regolith by siderophilic cyanobacteria to free up non-organic elements and create organic compounds for photobioreactor growth. Stage 2 will involve a photobioreactor with species of cyanobacteria that will be responsible for production of oxygen, fixation of carbon dioxide, and accumulation of biomass for use in human consumption and fuel for subsequent operations. Stage 3 will involve a third bioreactor responsible for the creation of biofuels (methane) for use in the DAV.

## 2. Human Living Requirements

**Figure 1:**
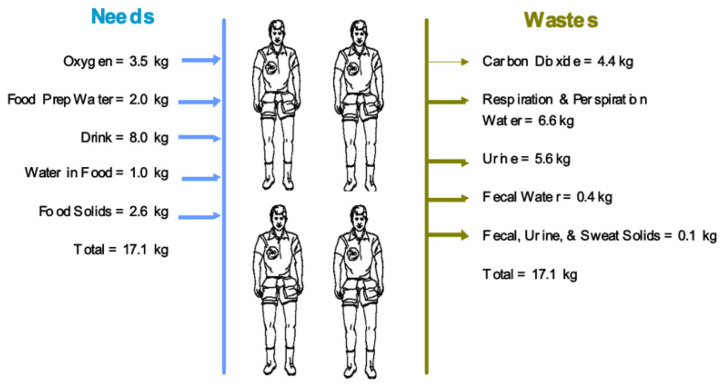
Inputs and outputs for a four-person crew [3].

The introduction of the human element to the mission is the basis for research into this field and establishes the need for generation of consumables and fuel during the mission. While we have developed technology and food packaging for efficient long-term storage and use of foodstuffs, oxygen reserves, and carbon dioxide scrubbing, using these for a mission duration longer than that of an ISS expedition requires a look into a more cyclical process. Instead of letting material fall into a sink of inert material, BLSS systems can complete the circle to reorganize human waste, combined with harvested materials, into usable consumables. To set a base for this discussion, we must discuss the expected consumable requirement of the human body in space. Ewert and Stromgren conducted a review of experiments and literature and constructed a frame for the metabolic mass balance for an 82 kg reference astronaut during long-duration missions. These values would vary with the size of the crewmember as well as the exercise regimen of the day with some days only completing the required exercises with the potential of other days requiring substantial movement and lifting while setting up experiments and SHAB duties [1]. For specific effects of increased activity among the crew, the 41 node METMAN model should be consulted. This is a model that divides the human body into ten cylindrical elements each composed of four compartments (core, muscle, fat, and skin), and the heat generation of each element is calculated [2]. Increased heat generation will result in the need for more kcal, necessitating more food, oxygen, and a larger reactor. This is one reason that would justify deploying a larger reactor than would be thought necessary. In total, a 3–4-person crew is expected to process 17.22–22.96 kg of consumables a day. The NASA DRA 5.0 provides a great illustration of the metabolic requirements and products of a 4 person crew in Figure 1 [3].

### 2.1. Atmosphere

The reference astronaut conservatively requires about 0.89 kg of oxygen daily for respiration, taking into consideration a need for 30 min of aerobic exercise and 60 min of resistive exercise a day [1]. That same average astronaut conservatively produces about 1.08 kg of carbon dioxide daily, taking into consideration the same exercise regimen. With the crew size of a long-distance mission, the scaled oxygen requirement and carbon dioxide production would increase to 2.67 kg/day and 3.24 kg/day, respectively, for a 3-person crew and then 3.56 kg/day and 4.32 kg/day, respectively, for a 4-person crew. BLSS systems provide the potential to decrease our reliance on mechanical means of atmospheric regulation. The current state of BLSS capability in this realm will be discussed in the Habitat ALS subsection.

### 2.2. Food

For this reference astronaut, daily food is estimated to measure at 0.80 kg dry mass before the addition of preparatory water. According to the FDA, the average human adult requires approximately 2000 kcal/day made up of approximately 78 g of fat (with 20 g of saturated fats and 300 mg of cholesterol), 275 g of carbohydrates, 2300 mg of sodium, 28 g of fiber, and 50 g of protein [4]. These numbers are different depending on the source used and vary to a certain degree depending on the size and sex of the person in question. Astronauts performing significant physical activity such as exercise countermeasures and planetary surface EVA (approximately 8 h EVA, 8 h IVS, and 8 h of sleep) will have additional caloric demands to maintain bone health, muscle mass, etc., of an additional 500–1000 kcal/day.

### 2.3. Water

The reference astronaut is also expected to have a daily requirement of 2.79 kg of drinking water in addition to the 0.50 kg needed for food preparation and the 0.76 kg that already exists in the food. This water is then expected to leave the astronaut in the amounts of 3.04 kg from perspiration and respiratory water, 1.40 kg in the urine, and 0.09 kg in feces. Some flight surgeons advocate up to 3 L/day/crewmember to reduce the risk of urolithiasis during prolonged periods of hypogravity exposure. It is not clear how much bone mineral preservation occurs with 0.16 or 0.3 g exposure on the surface of the Moon or Mars, so it is not clear what the risk of calciuria associated with bone loss will be.

Water is provided for missions through direct transportation, byproducts of fuel combustion, and reclamation of carbon dioxide through the Sabatier reaction [1]. Current theoretical estimates posit that about 5.33 kg of water can be recycled and made available for use while the human requirement is only 5.03 kg [1]. However, Ewert and Stromgren state that with the potential for loss through processes including towels for hygiene and disposed wipes, the margin is too small for comfort.

## 3. History of Life Support System Designs

### 3.1. Lunar-Mars Life Support Test Project

With a discussion of the future of life support systems, it becomes necessary to discuss the developmental history of these regenerative systems. The first development that we will review is The Lunar-Mars Life Support Test Project (LMLSTP), which was a multi-phase experiment conducted from 1995–1997 designed to evaluate the efficacy of human-in-the-loop, closed-environment life support systems in supporting crew habitation. These studies assessed the ability and interdependence of various air revitalization, water recycling, and environmental sensing technologies. Many of the lessons learned from these experiments were applied to the life support systems on board the International Space Station. In Phase I of the LMLSTP, researchers demonstrated that higher-order plants, in this case a wheat crop, were able to supply oxygen and remove carbon dioxide at such a rate as to support a single human for 15 days. Furthermore, it was demonstrated that the rate of photosynthesis in the crop, which is directly related to the rate of air revitalization, was able to be controlled through regulating photon flux, CO_2_ inflow, and optimizing growth conditions for the crop. In this fashion, it was demonstrated that the performance of regenerative life support systems can be controlled and regulated [5].

In phase II of the LMLSTP, four humans conducted a 30-day stay in a closed-loop environment. Air revitalization systems, utilizing Sabatier CO_2_ reduction processes, molecular sieves, and an electrolysis unit, were able to maintain oxygen levels between 20.3 and 21.4% and CO_2_ levels between 0.30 and 0.55% throughout the duration of the test [5]. Air filtration, accomplished by running the air through activated charcoal, reduced trace gas contamination to within acceptable limits. Water was recovered from shower, galley, laundry, urinal, and humidity condensate. Notably, this test represents the first time that NASA recycled water for potable use. Urine was processed through Vapor Compression Distillation (VCD), with a recovery rate of 98%. Ultrafiltration/Reverse Osmosis (UF/RO), and several post-processing systems, yielded a recovery rate of 95%.

In phase IIa, four humans conducted a 60-day stay utilizing ISS-like life support system hardware. The test utilized CO_2_ venting during the first 30 days, and CO_2_ removal as in phase II during the last 30 days. CO_2_ levels remained between 0.22 and 0.60% and O_2_ levels between 20.05 and 21.85% for the duration of the 60-day test [5]. All trace gas contaminants were within acceptable limits, with the exception of formaldehyde, which was present at a level of approximately 0.16 ppm throughout the test. The formaldehyde, the level of which exceeded spacecraft maximum allowable concentrations (SMAC) but was within the limit for industrial exposure, was found to be a result of off-gassing from acoustic tiles used in the construction of the module [5]. In this test, the water recovery system performed well but required servicing several times, with a total of five 0.5-micron filter changes, a replacement of the ion exchange bed of the Volatile Removal Assembly (VRA) at day 28, and a replacement of the Multifiltration (MF) unibed on day 45.

Phase III of the LMLSTP involved integrating the biological revitalization systems used in phase I with the physicochemical (P/C) life support technologies seen in phase II and IIa. A four-person crew was kept for 91 days in a closed-loop chamber, with a connected Variable Pressure Growth Chamber (VPGC) which housed a wheat crop. As expected, the plants produced an adequate supply of O_2_ and adequate CO_2_ removal to support one crew member, whereas the P/C systems provided for the oxygen and CO_2_ removal needs for the remainder of the crew [5]. In this phase, bioreactors were utilized in water recovery operations. These bioreactors utilized aerobic microbial species in order to oxidize carbon- and nitrogen-based molecules in wastewater. These bioreactors were integrated with the P/C system from Phase II and IIa. In this test, an initial eight-day supply of water was able to be recycled for the duration of the mission without any additional input of water into the system. This phase of the experiment also demonstrated the viability of a Solid Waste Incineration System (SWIS), which was able to incinerate crew waste to generate CO_2_, which was then utilized by the plants in photosynthesis [5]. Lastly, the crew was able to make bread with the wheat grown, as well as harvest approximately four heads of lettuce from a small supplemental growth chamber every 11 days in order to supplement crew nutrition. This supplementation, however, accounted for only a small portion of crew caloric intake.

### 3.2. BioHAB

There exists the idea that the turtle carries its home, the shell, while the bird flies out and builds its nest at its final destination. While carrying shelter is reliable, it is costly in energy and speed. This is a conundrum that must be solved when searching for a solution for habitation on either Martian or Lunar surfaces. The BioHAB project sought a solution for a light material that would still have the properties necessary to be supportive for Martian or Lunar operations. Fungal mycelium has been looked at as a biomaterial that is self-replicating, biodegradable, acts as an insulator, is fire retardant, and does not produce toxic gases like many plastics do. The compression strength is greater than that of lumbar and the flexural strength is greater than that of concrete [6]. Use of melanin-rich fungi can also absorb radioactivity to shield the astronauts from the increased radiation experienced on Martian surfaces.

In order to set up the dome that would be built, there would be a flexible plastic shell seeded with mycelia and dried feedstock [6]. Depending on the availability, the seeded mycelia would then be moistened with Martian or terrestrial water. It would be heated by focusing a Fresnel mirror or creating an exothermic reaction in the medium. When the structure is complete, the heating will be removed, and the mycelia will enter into a dormant phase. If additional structures or repairs are needed, the mycelium growth can be reactivated by adding back heat, water, and feedstock. It has been proposed that in addition to using mycelia to build the habitat, it can also be used following the same principles to build furniture and tools needed to sustain life [6]. The mycelia can be stained to look like wood which is proposed to have a psychological benefit for the astronauts.

Bacteria would serve an important purpose in this as well in 3 important manners. First, it would supplement structural integrity. It would, secondly, act as a biosensor to detect pressure and flaws in structural integrity by measuring the mechanical strain and reporting anomalies through either color change or fluorescence. Third, it would be able to provide metabolites to speed up the growth of the mycelium shell. The final layer of the shell would be an ice layer that would be created from Martian water that would serve as a protective layer but also would be able to be melted to be used for growth by the mycelium.

During multiple experiments done during NIAC phase I, different components of the proposed habitat were examined. First, it was estimated that only 781.5 kg of upmass would be needed for the bag and biology in order to build a reasonably sized habitat [6]. The material can be baked in order to kill the spores and add structural rigidity. When looking to see the optimal mycelia to use, it was seen that Ganoderma lucidum had the strongest material and grew quickly, but other species were more genetically modifiable. When growing the mycelium, it was observed that the moisture level greatly impacted the speed of growth. When allowed to get too dry, the mycelia would enter into the dormant stage but could then be reactivated with the addition of water [6]. It was confirmed that the mycelium is able to grow on Martian regolith simulant. One important observation was the impact of particle size on colonization rate as too large or too small of particles led to slower growth. Growth in various temperatures was observed with an optimal temperature of 30 °C, and dormancy was entered at temperatures close to 4 and 37 °C. Due to the natural temperature on Martian and Lunar temperatures, this confirmed the need to configure a heating system [6].

When observing the strength of modified mycelium, they were able to achieve strengths similar to that of Elmer’s glue demonstrating the capability of the mycelia to act as a glue [6]. In order to address the concern of filtered water to drink, mycelia were genetically modified to bind metals. When modified and used as filter strips, it was able to remove 92% of copper from water within 30 min demonstrating adequate filtration capabilities of metals.

Overall, the proposed strategy to construct mycelium habitats is to have an initial mission that would carry the plastic shell for habitats that are manufactured on earth and seeded with the mycelium and cyanobacteria [6]. Once at the destination, the habitats would be assembled with the help of robots on the scale of weeks. The cyanobacteria built into the layer of the shell would provide oxygen not only for the mycelia but also the inhabited area of the structure. It was tested and observed on a smaller scale model that it would theoretically take the cyanobacteria about 4 days to achieve 20% oxygen levels internally. Once the astronauts have arrived, the mycelium will act as their shelter, a medium from which to construct furniture and tools, water filter material, and also as food [6]. Human waste would be used as a compost and/or for power generation. Due to its ability to be reactivated and self-replicating, any additions or repairs could be sourced from current mycelium as opposed to requiring resupply missions as would be necessary for non-biological materials.

### 3.3. Micro-Ecological Life Support System Alternative (MELiSSA)

The Micro-Ecological Life Support System Alternative (MELiSSA) project, beginning in 1989, is a useful testbed for the technologies that must exist to operate closed-loop life support systems. Specific goals in designing the MELiSSA loop were to use robust microorganisms for production of O_2_ and nutritious biomass with direct use of light as an energy source, limited O_2_ consumption, compact reactor setup for space flight, and efficient re-conversion of minerals, waste, and CO_2_ [7]. The project established a pilot plant in 2009 with four distinct life support compartments: a liquefying compartment, a photoheterotrophic compartment, a nitrifying compartment, and a photoautotrophic compartment. In the liquefying compartment, waste is anaerobically converted into constituent molecules (CH_4_, H_2_, NH_4_) and volatile fatty acids, the latter of which is eliminated in the photoheterotrophic compartment. The sole function of the photoheterotrophic compartment is the elimination of volatile fatty acids, as these acids are ultimately toxic and unusable. The third component, the nitrifying compartment, utilizes the nitrogen-fixing bacteria *Nitrosomonas* and *Nitrobacter* in order to convert the ammonium produced in the liquefying compartment into nitrates, which can be efficiently utilized by plants. The final compartment, the photoautotrophic compartment, consists of a cyanobacteria reactor that uses Arthrospira platensis and a higher order plant component which provides for additional nutritional supplementation for the crew [8].

The MELiSSA project is, in actuality, a series of dozens of experiments to study the effectiveness and implementation of the compartments above. The MELiSSA project has gone even further to test these technologies in space; the ARTEMISS experiment aboard the International Space Station successfully demonstrated that a microbial photo-bioreactor was able to operate in a microgravity environment without any adverse effect on bacterial colony size [9]. Further, the program has generated numerous studies with valuable data that could be used in the implementation of full-scale closed loop systems in the future, including investigation into sustainable food production units such as tubers and wheat [10,11]. *Arthrospira* was found superior to trees in absorbing atmospheric carbon dioxide, as trees can fix 1–4 tons of carbon per hectare per year while cultivated *Arthrospira* can fix 6.3 tons of carbon and produce 16.8 tons of O_2_ per hectare per year. Hence, *Arthrospira* was the O_2_ producer and food supplement of choice in the conceptual design of the MELiSSA loop [12]. Lastly, the program developed protocols for urine and waste recycling [13], extraction of rare earth metals in space by biological processes [14], and urine nitrogen recovery techniques using membrane aerated nitrification [15]. These series of experiments provide a very useful insight into closed-loop systems, and the publications are too numerous to address the entirety of their findings in this manuscript.

A more recent consideration with regards to utilization of cyanobacteria in the MELiSSA loop is quorum sensing and the development of biofilms. Understanding the role quorum sensing plays in a closed life support system such as MELiSSA is important since active quorum sensing signals may serve as micropollutants that compromise other compartments. Currently, there are no studies reporting a quorum sensing communication system in *Arthrospira* sp. PCC8005. However, the cyanobacterium *Gloeothece* PCC6909 utilizes quorum sensing signals as does *Spirulina platensis* under conditions of stress. Interestingly, some cyanobacteria such as *Anabaena* possess quorum quenching genes than can serve a protective effect against such signals that may inhibit growth or have a toxic effect [16]. Some other studies as a part of the MELiSSA program will be cited throughout this paper.

### 3.4. Inflight Atmospheric Revitalization (ARV) Systems

Current atmospheric revitalization (ARV) systems that have been used on the International Space Station and previous spacecraft are worth considering and comparing to understand how atmospheric gases were handled. In general, ARV processes can be simplified into two fundamental categories—separations and reactions. Separations include physical adsorption, absorption, and mechanical filtration processes while reactions include chemical adsorption, oxidation, and reduction processes. In NASA’s Shuttle orbiters, separations- and reactions-based processes were used to remove CO_2_ from the cabin atmosphere, and trace volatile organic compounds were removed via separation processes. ARV systems have typically scaled with mission duration and crew size. Some of the technological approaches for atmospheric revitalization that were used historically on previous missions are shown in Table 1 below.

### 3.5. Solid Fuel Oxygen Generation (SFOG)

One type of atmospheric revitalization system that replenishes oxygen is the solid fuel oxygen generator (SFOG). The SFOG, also known as “oxygen candles” or “chlorate candles”, contains canisters with mixtures of dry particulate sodium chlorate (NaClO_3_) and iron (Fe). When the SFOG is ignited, the iron becomes combusted at 1112 °F (600 °C), which provides the thermal energy required for the following reaction:

NaClO_3_ (s) + Fe (s) → 3O_2_ (g) + NaCl (s) + FeO (s) (1)

Sodium chlorate breaks down into sodium chloride or table salt (NaCl) and oxygen gas (O_2_) while some of the oxygen combines with iron to form iron oxide (FeO) [17].

The SFOG system is designed to supply 6.5 man-hours of oxygen per kilogram in the mixture [17]. Each canister itself produces about 0.38 kg of oxygen, and 3–4 canisters can typically meet the requirements of a crew member relying solely on the SFOG system. Advantages of the SFOG system are that it provides a good source of oxygen gas resupply or supplementation and conserves water that would otherwise be used towards oxygen generation. For example, during a 6-month period on the international space station, it was found that an oxygen equivalent of 57.3 kg from SFOG resulted in 64.5 L of water savings, which amounted to 11% savings in the water used for oxygen generation over an entire year [18].

However, a limitation of the SFOG system is that it is accompanied by the formation of high temperature areas, which decreases both the efficiency and safety performance. A newer development from the SFOG called multicomponent nanostructured oxygen generators (NOGs) is based on a NaClO_3_–Sn–Co_3_O_4_ system and allows for reduction of the overall reaction temperature as well as elimination of hot temperature fluctuations [19]. Overall, while SFOGs may be a good backup source of oxygen, they do not appear to be a sustainable primary source of oxygen generation for long duration spaceflight due to the number of reagents and materials needed that are difficult to resupply.

### 3.6. Electrolysis

Electrolysis of water has largely been the primary method for oxygen generation in space. The NASA oxygen generating system (OGS) and Elektron (Russian electrolysis system) are two electrolysis-based systems that have been extensively utilized on the International Space Station. Water for both of these systems was primarily provided by Russian segment systems with back-up provided by U.S. resupply capabilities. Water regeneration occurred through the processing of condensate in the Russian water processor CPB K2M. Water is also recovered from a Water Recovery System (WRS) that is comprised of a Urine Processor Assembly (UPA) and a Water Processor Assembly (WPA) [20].

Water electrolysis in the Elektron-VM takes place through a pneumohydraulic mechanism. Alkaline electrolyte (25% KOH) is circulated through anode and cathode chambers by a circulating pump, and oxygen and hydrogen are produced which then leave the chambers as a gas/liquid flow. Gas/liquid flows pass through recuperative heat exchangers, coolers, and separators so that the liquid is separated from gas in static separators while the electrolyte is recirculated through the electrolyzer. The gases then pass through aerosol filters and a pressure control unit with the oxygen then supplied to the cabin atmosphere and the hydrogen being vented out into space, or potentially reused. 

The Elektron-VM system generates oxygen at a rate of 160 L/h and can support a crew of up to 6 people. Total power consumption by this system is typically in the range of 288–1344 W but may go up to 1500 W [21]. The NASA OGS system works similar to Elektron-VM by using processed water to generate oxygen within electrolytic cells. The OGS generates 270 L O_2_/cell per day and can collectively contain around 15 cells. In terms of capacity, the OGS can support up to 11 crew members [20].

The Sabatier reaction is worth discussing here as prior to its implementation on the international space station, any captured CO_2_ and the hydrogen (H_2_) gas generated by the water electrolysis systems were simply vented out into space. With the implementation of the Sabatier reaction and installation of the Sabatier system, the captured CO_2_ along with H_2_ generated from electrolysis could be used as substrates to synthesize water and methane (CH_4_) [22]. The Sabatier reaction is of great value since it can serve as a means of resupplying water. Methane is useful in fuel oxidation and propellants as well as in biofuel production involving bioreactors. Therefore, there is potential for synergy between water electrolysis and BLSS.

### 3.7. CO_2_ Scrubbing

With regards to CO_2_ scrubbing, both non-biological and biological options have been considered for the capture and removal of CO_2_ from the atmosphere. As was tested in the MELiSSA project and will be discussed later, algae provide a biological means for capturing CO_2_ and performing carbon fixation. Likewise, photosynthetic microorganisms also present a useful solution for capturing and fixing carbon dioxide. In this section, we will discuss several of the non-biological means of capturing and removing carbon dioxide from the cabin atmosphere.

### 3.8. Carbon Dioxide Removal Assembly (CDRA)

**Figure 2:**
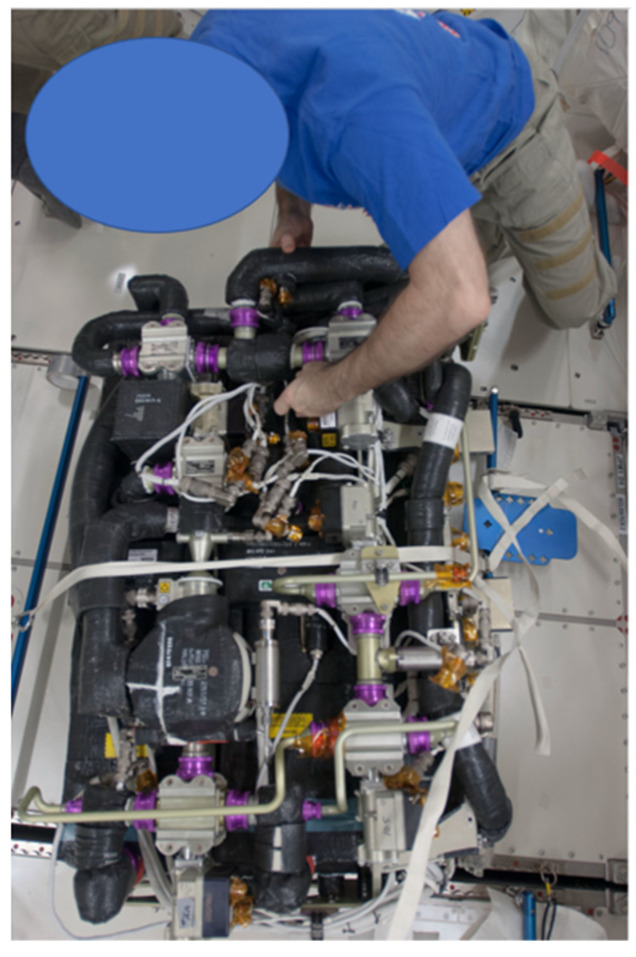
ISS crewmember performing maintenance on the CDRA (NASA Images Library).

The Carbon Dioxide Removal Assembly (CDRA) (Figure 2) was the primary CO_2_ removal system installed in the U.S. segment of the International Space Station. It operates as a dual-bed, zeolite-based CO_2_ removal system that utilizes an alternating cycle. There are two desiccant-adsorbent beds with one adsorbing CO_2_ from the cabin air while the other desorbs previously accumulated CO_2_ to space vacuum. As incoming air is first flowed over Bed 1, it is desiccated before being flowed over the zeolite portion of Bed 2 for CO_2_ removal. The beds alternate roles in a cyclical process when the adsorbent portion of Bed 2 becomes saturated with CO_2_. A pumping mechanism finally channels the CO_2_ to be vented or where it is potentially needed [23].

### 3.9. Vozdukh

Vozdukh was the primary CO_2_ removal system that was installed in the Russian segment of the International Space Station. Vozdukh operates on a principle that is similar to the one described for CDRA. However, the key differences for the Vozdukh system are that it uses three beds and an amine-based adsorbent rather than a zeolite [23].

### 3.10. Lithium Hydroxide (LiOH)

The lithium hydroxide method for CO_2_ scrubbing simply involves perforated cylindrical metal canisters filled with Lithium Hydroxide (LiOH) that allow for air to flow through the canisters. The canisters are placed in a blower system that provides motive airflow through both the can and the LiOH contained within. The LiOH reacts with ambient humidity and CO_2_ to create lithium carbonate (Li_2_CO_3_) with the conversion to Li_2_CO_3_ being irreversible. In other words, LiOH canisters are non-reusable and there must be fresh LiOH canisters supplied to continue scrubbing CO_2_ [23].

### 3.11. Metal Oxide (MetOx)

Similar to the LiOH canisters, MetOx canisters also work to remove carbon dioxide from the atmosphere. The biggest difference between the two of these systems, however, is that the MetOx system is regenerable [24]. Through an oven-baking process, trapped CO_2_ can be released and reintroduced into the atmosphere of the vehicle. This system is mainly used in the Extravehicular Mobility Units (EMU’s) to scrub out the carbon dioxide that the crew are exhaling inside these suits during EVAs, with the purpose being to save the gaseous resource while also preventing it from affecting the crew member in such a tiny environment. In the past, crew have complained about the foul odor that it gives off when baked, and it is believed that this is due to contaminants released. Air samples were taken in the past when this phenomenon happened and while contaminants were detected, they were all still within the normal limits of operation, and the crew were able to return to the U.S. segment of the station following atmospheric decontamination by the trace contaminant control system (TCCS) [24].

## 4. Habitat ALS

### 4.1. Stage 1: Resource Acquisition from Regolith

Cyanobacteria and microalgae require a variety of elements to employ in their biologic processes and without close proximity of resupply, it becomes necessary to address the gap in substrates using resources found on the surface of Mars. Martian regolith contains all of the elements that are needed by cyanobacteria to grow (C, H, I, N, P, S, K, Mg, Na, Ca, Fe, Mn, Cr, Ni, Mo, Cu, Zn, etc.) [25]. This fueled hope and inspired subsequent studies to determine whether terrestrial microorganisms can grow in the conditions present on the Martian surface. Each species will require different quantities of each element, but the overall collection is shared between species. While we have no direct access to true Martian regolith, investigators have shown that species of cyanobacteria successfully utilize both simulated regolith and atmosphere. Verseux et al. performed an investigation into the ability of *Anabaena* sp. to grow on a simulated regolith as well as in a controlled atmosphere of 96% N_2_ and 4% CO_2_ [26]. Their findings showed “vigorous growth” with the simulated conditions with an atmospheric pressure of only 100 hPa. Further, they also analyzed the biomass resulting from the *Anabaena* sp. growth and deemed it was a viable substrate for other BLSS modules to be employed due to its use by strains of *E. coli* [26]. The growth of *E. coli* using the *Anabaena* sp. Biomass as a substrate generated slightly higher mean biomass than use of a standard solution but was still within the margin of error [26]. *Anabaena cylindrica* was again deemed to be the ideal candidate for bioweathering of the basalt rocks present on both the Moon and Mars by Olsson-Francis and Cockell where it exceeded the growth potential of other tested species and showed that basalt can be weathered to release elements including calcium, iron, potassium, magnesium, nickel, sodium, and zinc [27]. Such a substrate can be used to power proposed modules used for generating fuels or other systems.

Another previous study confirmed the ability of *Nostoc* sp. to grow on a Martian regolith simulant. This simulant was based on the NASA-created JSC Mars-1 regolith simulant built from data from rover missions. In a simulated closed system at 90% of Earth’s atmospheric pressure, growth of the organism was exhibited [28]. Water was formed from the metabolic reactions and drops were observed on the inside of the glass [28]. It was concluded that the *Nostoc* sp. in this case had the ability to grow under conditions expected on Mars [28]. Of their more relevant candidates for food production, *Dunaliella salina* and *Chlorella vulgaris* were candidates for an investigation into viability of growth at lower atmospheric pressures again like that seen on Mars. Both organisms exhibited growth at pressure of 80 and 160 mbar with growth rates suffering in the lower pressure environment [29]. Due to the success, these species were used in a further experiment to test their growth in Martian regolith analogs, specifically Exolith MGS-1 solution, to which growth was shown in the *Chlorella* organism but not *Dunaliella* [29]. Further studies into these species will be performed using other simulants like the JSC-1. Genome sequencing of the variants successful in growing under low pressures will also be completed. Of note, in the same experiment, *Spirulina platensis* did not exhibit growth under the same low-pressure conditions [29].

Although some species have grown on regolith analogs, not all molecules in the regolith are available for biological use. Some necessary metals and compounds are trapped in basalt rocks and others that make up a good portion of the regolith. Further, Lunar regolith is relatively free of organic compounds, making it inhospitable to life [30]. Siderophilic cyanobacteria possess the bioweathering capability to free the useful compounds and elements needed from that regolith. Production of organic acids allow siderophilic cyanobacteria to break down the regolith into an aqueous phase for use in other applications [30]. The application applicable to this BLSS/ISRU system would be to use this aqueous phase of substrates, along with the biomass created in a Stage 1 reactor, to fuel the Stage 2 photobioreactor (PBR). Cyanobacteria have been responsible for supporting heterotrophic communities for billions of years, colonizing barren environments like deserts and creating the conditions for higher order life [25]. In their work, Brown et al. had identified a new siderophilic cyanobacteria species, named JSC-12, from their samples at Yellowstone that demonstrated the highest bioweathering activity among others, able to grow on samples made of 50% iron and titanium [30].

**Figure 3:**
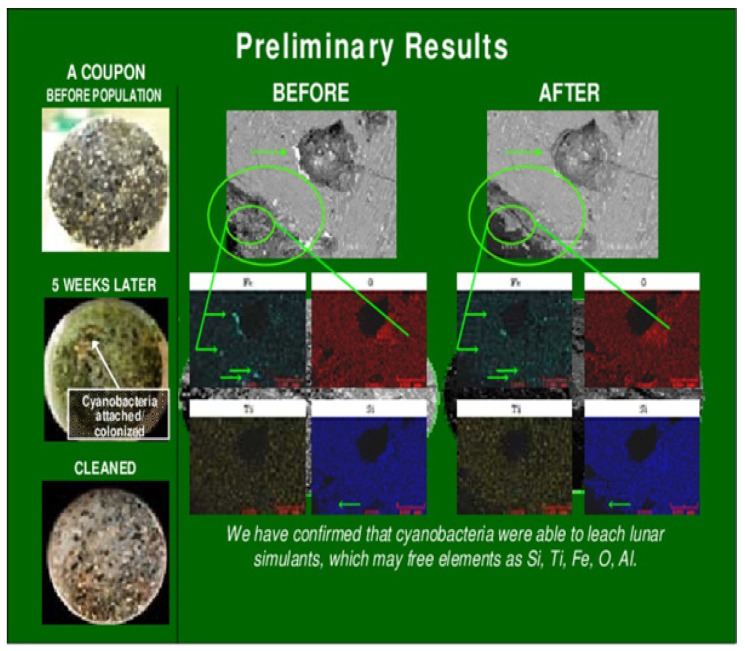
Bioweathering of Minnesota basalt grains by siderophilic CB JSC-12.

One significant quality of Martian regolith is the relatively high concentrations of iron. Iron is an important metal for human consumption, creating the building block for hemoglobin. *Arthrospira* sp. were tested for their growth while exposed to increased concentrations of iron in their medium. Increasing the concentration of iron in the growth medium to 2 mg/g improved the overall nutritional quality of the organism. Siderophilic cyanobacteria like JSC-12 showed higher growth rates in iron replete analogs of Martian and Lunar soils, but their metabolic rates decrease when that iron level is reduced (Figure 3) [30].

As will be mentioned in the biofuels section, a two-reactor system has already been proven with *Spirulina* biomass being fed to a methanogenic reactor. Work should continue on discovering the viability of siderophilic cyanobacterial biomass, and its aqueous solution should be fed to other PBRs to complete the utilization chain from regolith to consumables.

### 4.2. Stage 2: Biomass Production and Atmospheric Revitalization

#### 4.2.1. Atmospheric Revitalization

BLSS ISRU reactors will support the mission through performing biologically based CO_2_/O_2_ exchange, both from the exhaled CO_2_ from the crew and the large quantity of atmospheric CO_2_ on Mars. Cyanobacteria are thought to be the organisms responsible for oxygenating the planet billions of years ago and we aim to harness this ability to support our crew in inhospitable atmospheres. Current means of carbon dioxide scrubbing and oxygen generation are largely physiochemical. Each of these systems are described in the history section of this chapter and they all have their benefits and limitations. The biggest drawback is that they are not always re-usable. For example, once LiOH stores are deployed, there is no restoration of those canisters, depleting emergency scrubbing capability. The goal of BLSS reactors is to supplement or even replace these physiochemical systems.

Since oxygen evolution is a direct result of photosynthesis, maximizing photosynthesis will maximize oxygen production. For *Spirulina*, experiments have shown that the maximum oxygen evolution rate was 7.1 × 10^−3^ kmol O_2_ kg biomass^−1^ h^−1^ and occurred when the culture was grown at 36 °C, at a pH of 9.5, and a radiance of 130 W/m^2^ [31]. That value translates to 227.2 g of oxygen per kilogram of biomass per hour. Given that a 4-person crew needs 3.56 kg of oxygen a day, a design based on continuous generation of oxygen would necessitate about 0.65 kg of biomass to be functional. With the usual biomass density of open-air *Spirulina* cultivation ponds being 0.5 g/L, continuous production would at most need a 1306 L bioreactor just for replacement of inspired oxygen. Adjusting for an 8 h illumination period, a reactor three times the size of the previous calculation would be needed, meaning 3918 L. Of course, improvements in density of the culture will allow for smaller reactors, leading to less of a tax on the mass and volume limitations of the mission. For example, if the density of the culture is increased just to 1.5 g/L, then one could get away with a reactor that is 1306 L again, but for an 8 h illumination period. Each species of microorganism has its own oxygen evolution rate, and if better species than *Spirulina* can be found with the same benefits in its portfolio, then they could be considered for use.

The other side of the equation is carbon fixation. Like other photosynthetic organisms, cyanobacteria and microalgae take carbon dioxide from the atmosphere as the carbon source and use it to create energy-rich compounds. Carbon fixation is directly linked to biomass production, and about 1.8 kg of carbon dioxide is needed to create 1 kg of dry algal biomass [32]. Cui et al. achieved a carbon dioxide fixation rate calculated to be about 355 g/kg biomass per day [33]. Through the cyclical nature of photosynthesis, a culture that is producing the proper amount of oxygen to sustain a crew should be taking in enough carbon dioxide to scrub the air. With the Martian atmosphere containing about 95% carbon dioxide, we must be cautious about the exposure of our cultures to that level without proper experimentation. High levels of carbon dioxide have the potential to alter the pH of the culture, endangering the efficiency of organisms like *Spirulina* which grow in alkaline environments. Indeed, when cultures of *A. platensis* were subjected to Martian-like atmosphere of 100% CO_2_ at increasing pressures, growth rate decreased, and acidification of the culture intensified as higher pressures were introduced up to 500 mbar [34]. Growth rate of *A. platensis* also showed about a 20% increase in growth rate at 100% CO_2_ at 50 mbar over the culture growth in ambient air. Perhaps a solution to this acidification could be to have a supply of inert gas to mix with the Martian atmosphere that can be separated out again from the production of oxygen after photosynthesis happens; that way the pH can still be controlled, and carbon can be obtained from the environment.

One thing we must keep in mind in a closed system is the potential for the reactors to exceed the renewal rate for carbon dioxide supply. Imbalances such as this could lead to insufficient supply of carbon dioxide for continued metabolism. We must have a way to be able to control the rate of photosynthesis to be able to control the gas levels to match the needs and outputs of our crew members. Photosynthesis rates are often dependent on temperature. The ideal temperature for *Spirulina* is around 35 °C, and deviations from this cause its productivity to suffer [35]. Perhaps one solution for a throttling system would be to operate our PBR at less than ideal temperatures. Creating a large enough system that fulfills our requirements at temperatures lower than ideal would allow us the option to raise and lower the temperature, and thus gaseous evolution depending on the temporal needs of the mission. Of course, this would result in a reactor system far larger than what we would need to build while operating at ideal temperatures, but this would provide a new level of redundancy with size and further modularization.

#### 4.2.2. Food Generation

Stage 2 of the BLSS/ISRU system is the creation of consumables. The goal should be to safely provide as much of the daily requirements for the crew through BLSS/ISRU as possible, thus reducing the mass of the transit vehicle and lengthening the potential duration of the stay on the planet. It is widely accepted that most of the dry weight of many microorganisms will be composed of proteins, while carbohydrates and fats lag. This composition does not directly line up with the human daily intake. Specifically, the high protein content of many microorganisms is cause for concern during long-duration dependence due to the risk of developing high uric acid levels, leading to development of gout or uric acid stones [36]. Both conditions could endanger both the mission success and the lives of the crew. Experiments to increase cyanobacterial carbohydrate content to necessary levels have had varying success. The highest carbohydrate content in a wild type cyanobacteria was 28.4% in *Scytonema bohneri* [36]. To alter the macronutrient composition of *Chlorella*, introduction to nitrogen-limited environments increased the carbohydrate content of the organism, but there is conflicting evidence surrounding the exact amount. One study showed an increase to 65% overall carbohydrate composition while another showed only a 10% increase [36,37]. Studies on *Spirulina* showed that by reducing nitrogen concentrations from 400 to 20 mg per liter, carbohydrate content of the culture grew to 74 ± 3% without affecting the total biomass yield [38]. In fact, before the nitrogen stress, the control culture carbohydrates were dominated by rhamnose (30.1%), glucose (30.9%), and galactose (21.4%), whereas within the nitrogen stressed cultures, the carbohydrate content shifted to the point that glucose made up 83.2% of all sugars [38]. If certain strains of *Chlorella* sp. and *Spirulina* sp. can increase carbohydrate yield in times of nitrogen limitation, then the relatively low nitrogen environments of the Moon and Mars may beneficially alter the composition of *Chlorella* or other organisms to match human nutrition more closely. 

Higher carbohydrate concentration also has a benefit in the harvesting process. Carbohydrate-rich *Spirulina* demonstrates a higher specific density which leads to faster settling rates of the culture in an un-agitated tank, the maximum rate being 0.64 m/h [38]. Gravity alone drives the separation of these cultures. Of course, these experiments are done with the gravitational pull of Earth and so the resulting settling rate on the Moon and Mars is altered by their unique gravitational forces (16.6% and 37.5% that of Earth respectively. Equipment-wise, this opens the possibility of using a lamella separator for harvest instead of just a centrifuge. In fact, one square meter of a lamella separator is said to be able to remove 94% of the water from 1 ha of open pond culture with only gravity [38].

**Figure 4:**
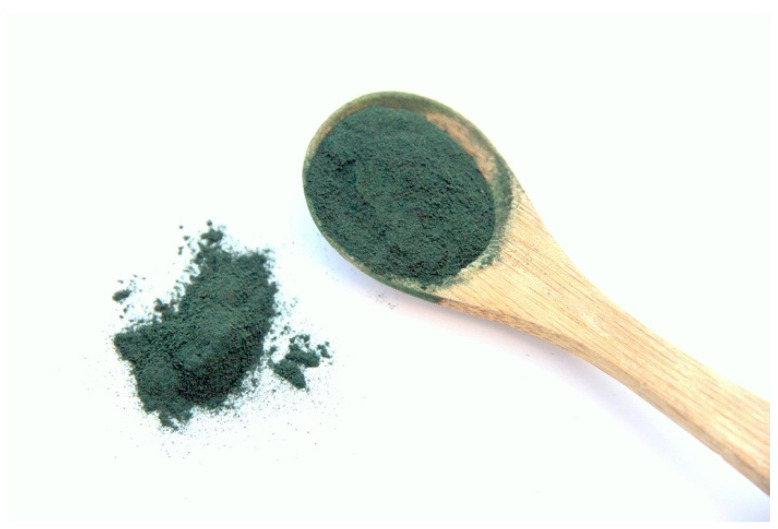
Dried Spirulina powder.

Each species of cyanobacteria and microalgae provides a unique composition of nutrients. Even then, different strains of each species have been shown to differ slightly from one another. A few species have gained popularity in cultivation in recent decades and have been studied for human consumption in a variety of realms. A few of the species of interest which we will mention in this section are *Arthrospira*, *Chlorella*, *Euglena*, and *Dunaliella.* Perhaps the most widely adopted is the genus *Arthrospira* (*Spirulina*) (Figure 4). Every 100 g of dry mass contains over 350 kcal, 4 g of fats, 17 g of carbohydrates, and 60 g of proteins [39,40,41,42]. While the fats contained in *Spirulina* are essential to humans, including linoleic and gamma-linolenic acids, the fat content overall is considered low and should be supplemented with other sources including seeds and nuts [39]. The same should be said about the carbohydrate content of this organism. Low relative carbohydrate content does not match with the human diet. Of note, toxic heavy metals present in the species have been deemed not to exceed EU regulations for consumption [43].

*Euglena* sp. offer a similar nutritional profile, boasting 39–61% protein, 14–18% carbohydrates, and 14–20% fats [44]. It also has a high crude protein efficiency, leading to fewer digestive losses as methane [45]. *Chlorella* sp. first became popular in east Asia, containing 57% proteins, 6% carbohydrates, and 17% lipids. Though the overall fat level is higher than *Arthrospira* in this case, it was noted that the types of fats included fewer essential ones including stearic, linolenic, and arachidonic acids [46]. *Dunaliella* sp. are proven to be cultivated on a large scale and have qualities that make it stand out over the other organisms. Namely, these organisms have one of the highest carotenoid contents of any species, pointing to a benefit in radiation protection of the astronauts. It also lacks cellulosic cell walls, allowing better digestibility [47]. Like other microorganisms, *Dunaliella* has high protein and low-fat contents and has been shown to increase carbohydrate content in times of nitrogen stress [48]. It has not been used for dedicated nutritional supplementation to date, and thus no reliable nutritional analyses have been done; however, it has been used pharmacologically for treatment of hypertension, atherosclerosis, Crohn’s disease, bronchodilation, and photo damage [49].

Overall, optimal growth conditions and maximal growth rate differ for each organism. The maximum specific growth rate of *Spirulina* organisms is around an 87% daily increase in biomass [50]. Culture conditions to achieve this include an irradiance level between 332 and 465 μmol photons m^−2^ s^−1^, temperature ranging 30–35 °C, and Zarrouk’s medium, either pure or 50% diluted [50]. Fortunately for the goal of recycling elements, *Arthrospira* was found to prefer urea over nitrite and nitrate as a nitrogen source up to 120 mM, allowing human waste to feed Stage 2 [51]. Another condition to consider for these reactors is the pH requirement for different organisms. Harsher pH environments reduce the ability of many organisms to grow, but many cyanobacteria and microalgae have developed the ability to flourish in these extreme environments. *Arthrospira* sp. tend to prefer alkaline environments (ideal pH 10.5), and *Euglena* sp. can grow in acid environments as low as 3.5 pH (as opposed to *Chlorella* sp. and *Dunaliella* sp. who lack this ability) [42,47,52]. This substantially decreases the chances of contamination of the culture by other, more sensitive bacteria who prey on these organisms. Invasive organisms are not always as adaptable. Although we have yet to find living organisms on Mars, there is the possibility that they exist. Since we are planning on using Martian regolith to power this reactor, any life in the regolith could contaminate the culture. Cultivating a species that can survive in life-limiting environments protects our reactor not only from bacteria that we inadvertently introduce ourselves but also from possible invaders from the regolith we introduce. Of course, if our selected culture species have evolved to survive conditions on Mars, then that can’t exclude other native organisms from being capable of the same. 

The most important logistical detail is the size of the reactor needed to fulfill all the requirements of a mission. Estimates and experiments have shown that in the current open water cultures for mass production, there exists about 0.5 g of dry weight in every liter of culture when growing *Spirulina*. Given the Caloric composition of over 350 per 100 g of dried *Spirulina,* if, hypothetically relying solely on *Spirulina* nutrition, we were to plan to get 3000 kcal out of this reactor for each of the expected four crew members every day to account for the extra activity while on the planet, that would equate to approximately 862.5 g of dried biomass per crewmember or 3.45 kg overall. A reasonable assumption based on the optimal growth rate would be the desire to leave 60% of the culture in the tanks to grow further and not deplete the culture. This calculates to 17,250 L of viable culture in a reactor before taking into consideration the need for redundancies and extra biomass to fuel Stage 3 of the ISRU system. Redundancies and preparing for turnover crew size will be discussed in a later section.

These calculations will be altered with increased productivity of cultures leading to higher densities. Considering the biomass density of 1.77 ± 0.02 g/L achieved in the experiments done by da Fontoura Prates et al. when they tested different wavelengths of light on *Spirulina*, the total calculated size of the necessary reactor would be around 4875 L, conserving mass and volume [53]. Thus, part of the preparation for these missions should involve the determination of the specific strain of microorganism to be used, take these ideal growth conditions into account, run trials for that strain, determine the growth rate, and design a reactor sized appropriately to that number.

An existing concern about microorganism consumption is the risk of toxins that many species create, including microcystins, nodularins, saxitoxins, and anatoxins [39]. A wide variety of incapacitating symptoms are possible including nausea, vomiting, pneumonia, hepatic failure, dermatitis, blisters, and kidney pathologies [39,40,54]. Fortunately, *Spirulina* and *Chlorella* are widely understood to be free of these harmful toxins and rendered “Generally Recognized as Safe” by the FDA [25,55]. *Euglena* strains have also demonstrated safety, passing acute toxicity, subchronic toxicity, and teratogenic studies [55]. High concentrations of Vitamin A in *Spirulina,* while still an essential molecule, have introduced limits to the amount able to be consumed in a day [25]. This will have to be addressed through strain selection or genetic engineering before relying heavily on this organism. It should be kept in mind as well that with the introduction of a Stage 1 bioreactor that feeds biomass into Stage 2, the species used in Stage 1 should not produce these toxins either, as they will inevitably make their way into the diets of crew members. Ultimately, preventing contamination of a bioreactor will be the best defense from toxins, and taking advantage of growth of our preferred species in exclusionary environments would be the avenue to do that.

### 4.3. Stage 3: Biofuel Production

**Figure 5:**
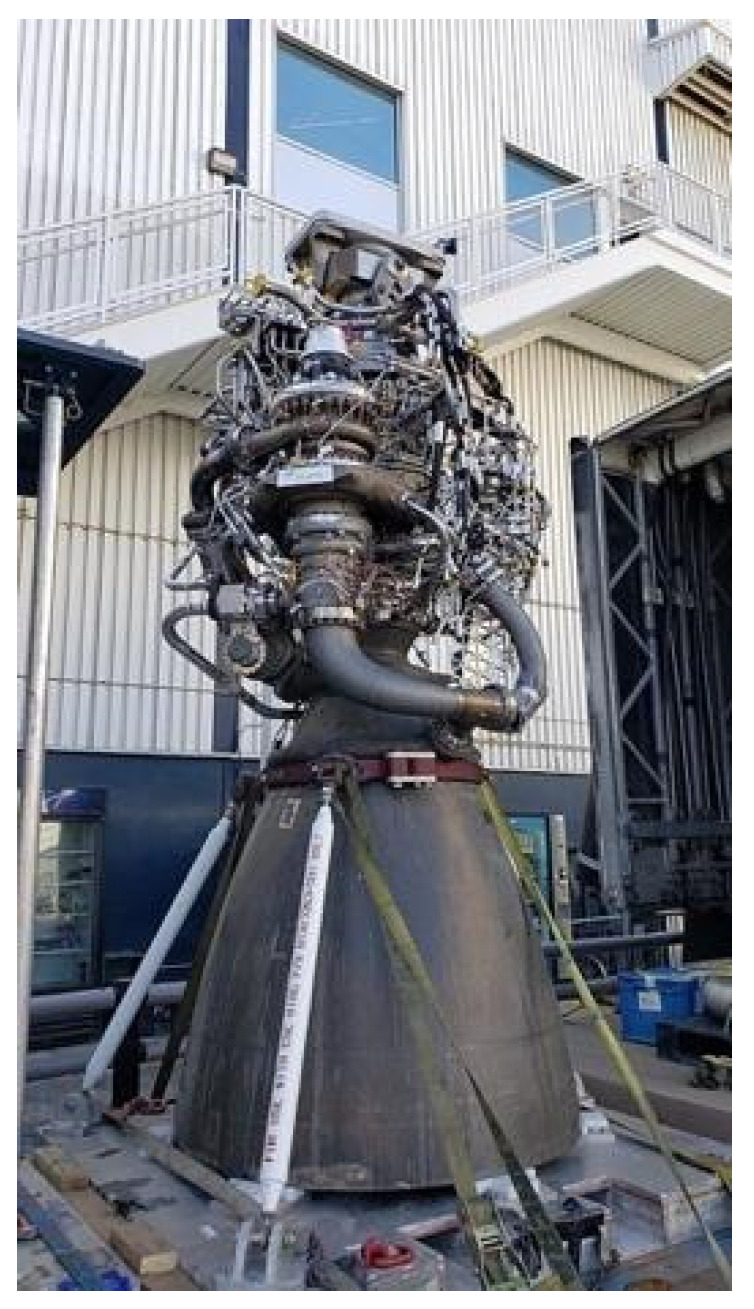
SpaceX’s raptor engine, one of the first widely used LOX/methane engine [57].

ISRU production of fuels for mission use is critical to maximizing efficiency. This will be accomplished by Stage 3 of the proposed design. Without a source of new fuel generation, launching all the anticipated propellant with the primary mission stack is a heavy tax on the allowed mass. LOX/methane, LOX/ethanol, and LOX/H_2_ engines are the current ideal considerations for missions to other celestial bodies because these chemicals have the potential to be produced from native materials. They each have their advantages and drawbacks. LOX/LH_2_ is the current fuel of choice for NASA and will be used for the Space Launch System (SLS) vehicle but new advancements in rocketry are allowing a variety of fuels to be adopted. Of the 3 fuels mentioned, LH_2_ has the costliest storage requirements, needing to be kept around 23 K, whereas methane needs to be kept around 100 K (still below the lowest lunar night temperatures), and ethanol can be stored from 150 K to 350 K [56]. Further, LOX/LH_2_ boasts the highest Isp out of the three, with LOX/methane coming in second and LOX/ethanol trailing, but it is noted that the use of LOX/LH_2_ in any kind of lander or ascent vehicle increases the complexity dramatically due to complex handling requirements [56]. LOX/methane has historically been proposed as the most suitable for Martian production due to the availability of CO_2_ in the atmosphere while LOX/LH_2_ has been preferred for the Moon due to relatively lower concentrations of those atoms. However, is it noted that it is still possible to produce methane on the Moon due to solar wind depositing carbon and other volatiles [56]. The two biggest noted drawbacks for methane adoption are the production rates and storage requirements. LOX/methane is in the beginning stages of adoption into the mainstream of rocket fuels, with SpaceX’s Raptor engine being a prime example (Figure 5). Hydrogen production by cyanobacteria has been demonstrated but at this point in time is not high enough to be practical for ISRU applications [25]. Overall, methane is a much more attractive solution due to the low density of LH_2_ and increased complexity of LH_2_ infrastructure among other reasons [25]. Methane is a cleaner fuel source than kerosene, eliminating the residues left behind after burning, therefore leading to higher reusability. ISRU reactors are the current projected means to provide this fuel, and there is plenty of data to suggest that production of oxygen and methane by microorganisms can provide for an ascent stage. For this section, we will follow with the historical proposals and discuss the production of methane for future BLSS/ISRU systems. 

We must first determine how much oxygen and methane are required for the ideal combustion and propulsion. Methane molecules have a molecular weight of 16 amu whereas oxygen has a molecular weight of 32 amu. The equation for combustion of methane is shown below. Stoichiometrically, four times the mass of oxygen as methane would be needed. However, in practice, LOX/CH_4_ engines are run with fuel on the leaner side of the spectrum. This ensures a few things, including that unburnt oxygen does not damage the engine, and there are some molecules available to create the thrust through their ejection velocity. This phenomenon was shown in a review of LOX/HC fuel combinations in which the theoretical mass-specific vacuum impulses of multiple propellants were calculated. The study did center their calculations around the vacuum environment, and it showed LOX/Methane was calculated to have its I_vac_ peak at a value slightly less than a 4.0 ratio of LOX:methane, pointing to the ideal fuel being leaner than stoichiometric proportions [58]. Indeed, Elon Musk confirmed a tweet that the propellant used in SpaceX’s Starship Raptor engines will be 78% LOX/22% methane, a 3.55:1 ratio [59].

CH_4_ + 2O_2_ → CO_2_ + 2H_2_O (2)

Methane Combustion.

PBR production of oxygen will be left for the previous discussion of atmospheric revitalization as the adjustments to provide extra oxygen for propulsion would just involve scaling up the existing production. Liquid oxygen is separated from the air through liquefaction of the atmospheric air with cryogenic distillation to follow [60]. On earth, the carbon dioxide, water, and hydrocarbons are removed before liquefaction leaving the oxygen and nitrogen to be separated. For use on Mars, the gas being put into the separator would be the internal atmosphere of the bioreactor system rather than the outside atmosphere. Oxygenated gas would be siphoned out of the enclosed reactor system and replaced with more Martian atmospheric carbon dioxide to allow photosynthesis to continue. Storing this cryogenic liquid will be a challenge of its own. As will also be stated in the prepositioning section, relying on the shaded areas on the Martian surface is not a viable option for passive cryogenic storage since even the shaded sections reach upwards of 35 °C and oxygen’s boiling point is −183 °C [61]. Instead, powered storage applications will be needed, further taxing the mission. This power could be achieved through any of the means previously discussed; however, reliability is key for this component as improper maintenance could lead to liquid loss and leave the DAV with no fuel. In that vein, RTG technology should be preferred over solar arrays due to their all-weather production as well as combined electric and thermal generation. There are a few systems designed to hold liquid oxygen long-term. Dewars are tanks that range in size from 5–200 L, cryogenic liquid cylinders range from 80–400 L, and cryogenic storage tanks usually range from 500–420,000 L [60]. During the shuttle era, NASA stored liquid oxygen in a 900,000-gallon tank near the launch pad [62]. These tanks are insulated from heat transfer and are constantly monitored for changes in pressure so that adjustments can be made for stability. Current cryogenic storage tank designs also have the capability to link to the vehicles, so fuel does not have to be stored in the DAV during deployment. However, storing some fuel in the DAV could provide mitigation for risk of tipping over as was feared in the novel/movie *The Martian*? Martian weather and dust storms can become formidable events with winds reaching upwards of 70 miles per h [63].

Due to contracts for the future HLS system only recently being discussed, we do not have the specifications of the expected lander for estimates of the amount of methane needed to be created by ISRU reactors. The average acceleration due to gravity on Mars is 3.71 m/s^2^ and the average on the Moon is 1.62 m/s^2^, about 37.8% and 16.5% of that of Earth [64,65] Air resistance will also not be as significant on Mars and certainly not an issue on the Moon when compared to the Earth. The Martian atmosphere provides just 6.1 hPa of average surface pressure [66]. Estimates have been done to say that we would need between 30 and 40 T of LOX-methane fuel to achieve the delta-v of 4.1 km/s needed for a launch vehicle from Mars to orbit, with the same vehicle requiring only 10 T to launch from the Moon [67]. For the scope of this paper, we will only discuss producing the fuel to get to Low Mars Orbit (LMO) as the fuel to be used by the transit vehicle could be already aboard. Assuming a 3.85 ratio of oxygen to methane needed to achieve successful flight from the surface and using the higher estimate of fuel needed, 31.75 T of LOX would be required alongside 8.25 T of methane. 31.75 T of LOX equates to about 25,230 L or 6670 gallons, and 8.25 T of methane equates to about 4253 gallons or 16,096 L. This requirement would fit well in currently available storage infrastructure here on Earth, but we would need to get this infrastructure to the Moon and Mars.

**Figure 6:**
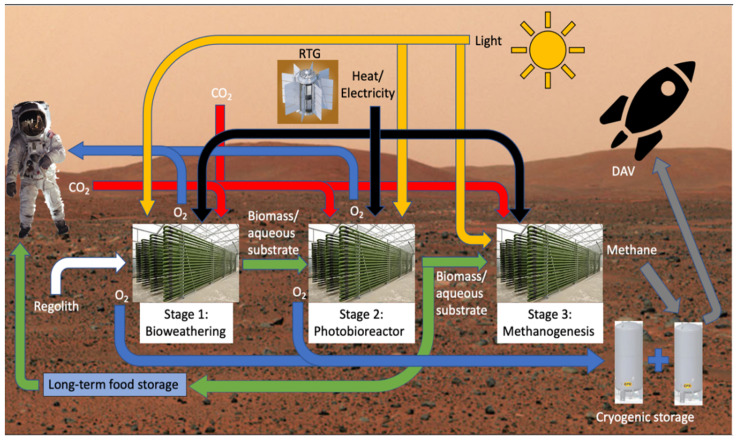
An original schematic for the interaction between the Martian environment, crew, and 3-Stage BLSS/ISRU system.

Methane production will be the third stage of our ISRU system, taking in biomass from the Stage 2 PBR. At present, the state of research into commercialized methane production by bacteria is in its early stages, with optimization still being worked out. However, experiments have been done that verify the use of photosynthetic cyanobacteria as food for a methanogenic reactor. *Spirulina* was fed to a reactor populated by *Methanocalculus* sp. which then also used carbon dioxide and formate to produce a methane content of 92–96% in its biogas [68]. The tradeoff experienced was that this high relative production of methane was met with low overall biogas production. A lot of the methanogenic organisms investigated come from the archaea domain and are not as adaptable as cyanobacteria. Protein-rich diets lead to increased ammonia levels in the culture which inhibit methanogenesis [68]. Slow rates of methane production in current designs could benefit from the evidence for increased carbohydrate content in cyanobacteria through nitrogen stress. Feeding the Stage 3 reactor with the same carbohydrate-rich cyanobacteria biomass that the crew eats might lower this inhibition. If ammonium inhibition cannot be avoided, then bioaugmentation with *Methanoculleus bourgensis* has also been shown to increase methane production by 31.3%, but this concept has not been tested in reactors fed with cyanobacterial biomass [68]. Breaking down cyanobacterial cell walls with thermal pretreatments at 100 °C for 8 h had shown a 33% increase in biogas productivity over the untreated biomass [69]. Indeed, 60% of untreated biomass will remain undigested. As for specific production rates, methane yield from microalgae loading ranges from 24 mL g^−1^ VS to 800 mL g^−1^ VS. One resource reported that different experiments showed production rates of 204.1 and 492.8 g of methane per liter per day of continuously stirred reactors using *Methanobacterium* sp. [67]. For a 300-day pre-deployment mission to create 8.25 T of methane with the more conservative production rate of 204.1 g per L per day, a reactor of approximately 125 L would be required. Of the possible organisms to be used for the Stage 2 PBR, preliminary results compiled in a review showed that *Spirulina* biomass can be digested to make 481 mL g^−1^ VS, *Chlorella vulgaris* at 403 mL g^−1^ VS, *Euglena gracilis* at 485 mL g^−1^ VS, and *Dunaliella* at 440 mL g^−1^ VS [69]. Another major problem with this field of research is that comparison of production rates cannot be directly compared between experiments due to lack of standardization in the terminology [69]. More work is needed in this field to gain the knowledge needed for effective hydrocarbon fuel production for ISRU dependent DAVs. The summary of a 3-stage system is shown in Figure 6 below.

## 5. Mission Planning Considerations

### 5.1. Illumination

Like all photoautotrophs, cyanobacteria require solar radiation to complete their photosynthetic processes. In their natural habitats, Earth’s own solar cycle provides periods of stimulation and rest to these organisms. Photobioreactors exist in 3 states with respect to irradiance. Photosynthetic production increases with irradiance up to a maximum rate (P_max_) at light saturation (I_S_), at which point the photosynthetic rate plateaus, until reaching light intensity high enough for photoinhibition (I_h_) [70]; 24 h sunlight deprived cyanobacteria of time to perform protein synthesis and respiration [71]. Every species of photosynthetic organism has a unique optimal irradiance level and wavelength, and therefore, the irradiance allowed in the system will have to be designed after selection is made.

Just as in any swamp pond with algae growing on the surface, algal cells closest to the light source multiply at higher rates and create shading conditions for those under them. In a BLSS/ISRU reactor, this same phenomenon can come into play with peripheral biomass shading the reactor core. There are two main solutions to this: increase the circulation of the culture or reduce the depth. On Earth, it is known that decreasing the depth of culture ponds achieves greater biomass densities [71]. Tubular reactors are believed to be an advantageous design, allowing 360-degree illumination, though shading can still be a problem if the tube diameter is too large. For this reason, tube diameters are usually set around 10 cm [72].

**Figure 7:**
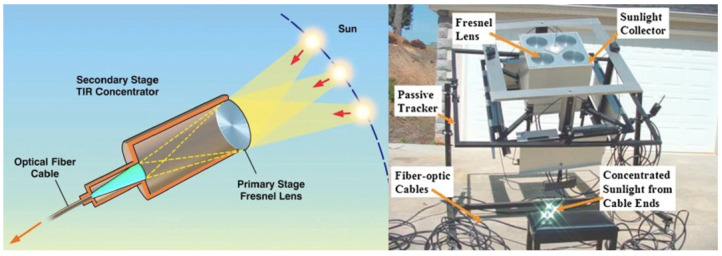
Basic design of a solar light concentrator to fiber optics (**left**) and an existing prototype designed by Gorthala (**right**) [73].

There are multiple methods of light delivery systems ranging from sunlight to halogen lamps to LEDs, each with their benefits and drawbacks. Fiber-optic cables provide the ability to direct sunlight from a parabolic solar device into the photobioreactor. Through this apparatus and the use of distributor plates, photons can be directed from the sun straight to the reactor, covering as much area as possible with a light source that contains all parts of the visible spectrum [70]. Optical fiber cables boast the ability to be sterilized as well as survivability against known culture agitation methods [72]. While cable survivability is noted, some drawbacks are that heat-based sterilization is not always possible depending on the ISRU design and cell adhesion to the fiber may lead to ineffectiveness of the agitation to distribute light to the whole culture. The loss of light in some fiber cables must be evaluated to find a suitable candidate for the design [72]. A design for a solar light concentrator system can be seen in Figure 7 [73].

When compared to fiber optics, external light sources (halogen and tungsten filament lamps) resulted in higher yields, thought to be due to reduced irradiation of the fibers [70]. However, when they were all used in unison, the resulting yield continued to grow beyond any individual source use [70]. Carvalho et al. compared the external light sources, showing that LEDs have by far the longest life span, with up to 50,000 h. They also give off most of their energy in the 600–700 nm range. Halogen lamps are low-cost, low intensity sources that also convert a significant amount of their energy into heat. Incandescent bulbs are the cheapest of all the sources and have the shortest life span. Fluorescent lamps have a higher intensity than halogen and incandescent bulbs as well as give off more of their energy in the 400–500 nm and 600–700 nm ranges (25.0% and 20.7%, respectively). They convert less energy to heat than halogen and incandescent but still do not provide the high intensity and heat reduction of LEDs [70].

Each species of photosynthetic organism has its own preference of wavelength for optimal growth. While whole spectrum light is often used for the outdoor cultivation ponds and would be used through the fiber-optic system we have discussed, investigating which specific wavelengths are used could allow us to tailor our artificial light sources to provide light to maximize productivity. With any photosynthetic organism, the two main light spectrums of interest are blue (450 nm) and red light (660 nm). *Synechocystis* sp. PCC 6803 exhibited a five-fold decrease in productivity in blue light compared to red at low intensities, but that difference decreased when intensity was increased [74]. *Arthrospira platensis* displays its most efficient biomass productivity around 620 nm [75]. One theory is that blue light creates an imbalance between the photosystems of the organism. PSI:PSII ratio decreases when exposed to blue light because the blue light is not absorbed as well by phycobilisomes. Phycobilisomes are a larger component of the photosynthetic system of cyanobacteria compared to other plants and algae. The relative rejection of blue light by phycobilisomes would explain the naming of this group of organisms “blue-green algae”. Further, the specific growth rate and oxygen production of *Synechocystis* increased with increases in red light concentration up to 60% red light [74]. High carbon dioxide conditions, as will be experienced on Mars, are also associated with decreased PSI:PSII ratio, leading to higher utilization of blue light than in conditions with Earth atmosphere. *Spirulina* was also put through trials of growth in different wavelengths. Results indicated that when grown under red light exposed integrally, the cyanobacteria exhibited a growth rate twice that of the control under full fluorescent light, leading to a culture density of 1.77 ± 0.02 g/L [53]. Intensity is another factor to consider in maximizing growth conditions. Kumar et al. showed that growth of *Arthrospira platensis* under a 12 h light/dark interval is maximized when exposed to 2000 lux when using fluorescent light, with further increases in light intensity leading to higher carotenoid content [76]. Other studies have shown that light intensity should be over 180 μmol photons m^−2^ s^−1^ in order to be effective at stimulating growth and that the maximized intensity is somewhere near 500 μmol photons m^−2^ s^−1^ for *Spirulina* [52,77]. Farges et al. demonstrated that the use of high efficiency red light does not offer a difference in biomass productivity from the control white light given that the same number of photons were introduced [75]. Other minor notes, green light stimulated the highest phycocyanin concentration at 2.8 times the control, and green, red, and white LEDs stimulated increases in fatty acid concentrations [53].

With the evidence described, a tubular or flat panel design combining fiber optics as well as exterior lighting would be the best setup for a mission critical reactor. Not only has a combination of light sources shown yielded higher productivity, but also this would provide a level of redundancy during times where sunlight is not available.

### 5.2. Power Generation

ISRU systems, like any other, need a source of power, whether that be thermal, electric, or solar. Efficient photosynthesis requires a balance of temperature, airflow, nutrient availability, and solar radiation. With the currently accepted assumption of a 300-day production timetable before crew arrival, estimates from the latest DRA convey an estimated power requirement for consumable ISRU at 2 kWe from continuous generation and 6 kWe from an 8 h/day solar cycle [3].

**Figure 8:**
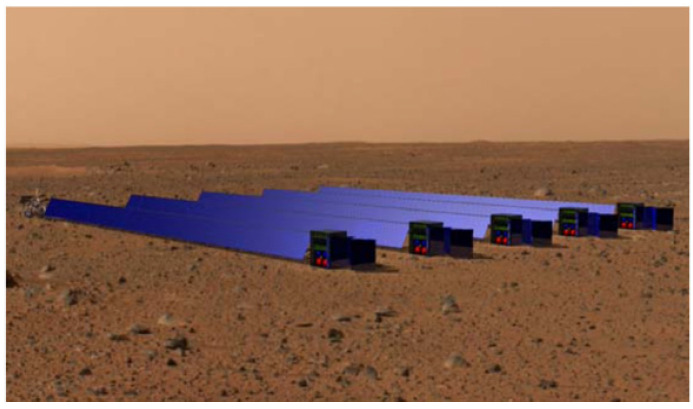
Illustration of Solar Arrays positioned on Mars [3].

The NASA Mars DRA describes a few existing power sources that could be used to generate the needed energy, the first being a modular solar array system. The current solar design includes a set of five, 5 kWe, 1980 kg modules, four for nominal requirements and one for redundancy (Figure 8) [3]. However, the report also states that an extra 450 m^2^ or 2500 kg of solar array would be needed to accommodate both the consumable and propellant ISRU. However, solar arrays are at the mercy of dust and weather. Since the power source is external, any obstruction of the solar cells would halt peak electricity generation.

**Figure 9:**
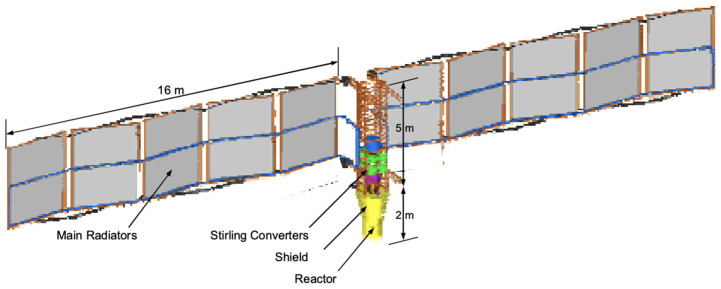
Concept image of a Lunar Fission Surface Power System [3].

Another proposed system is a fission surface power system (FSPS) (Figure 9) [3]. This system is touted for its low-cost, low-energy design and adaptability to multiple architectural designs. It can be made into different sizes, with the DRA stating that a 20 kWe, 6800 kg reactor could power the consumable ISRU and a 30 kWe, 7800 kg reactor could supply the propellant ISRU [3]. Current systems are already scalable to handle this with the minimum power generation being 10 kWe, with the ability to scale to 40 kWe [78]. This system would not be affected by solar cycles but would be a substantial radiation risk to the crew. Even with substantial shielding, it is proposed to have the FSPS stationed no less than one kilometer away from the HAB, with the crew dose rate of <5 rem/year.

**Figure 10:**
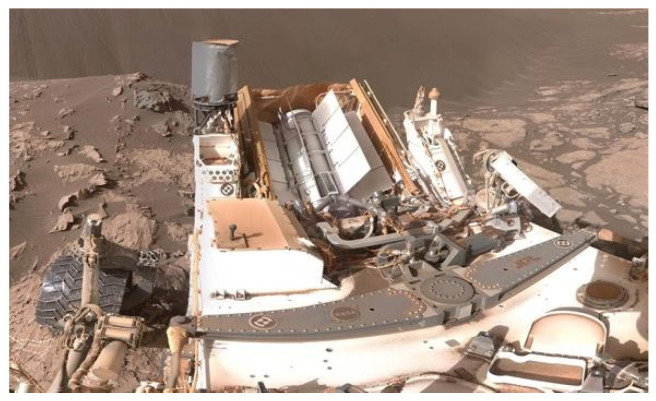
Multi-Mission Radioisotope Thermoelectric Generator on the Curiosity Mars Rover [79].

A third system is a radioisotope power system (RPS), sometimes referred to as radioisotope thermoelectric generator (RTG). Like the FSPS, RTGs constantly generate power, and these systems are already in use in probes and provide constant power generation through the decay of plutonium-238. The *Curiosity* rover was the first rover to be equipped with an RTG which has allowed *Curiosity* to work beyond its intended lifespan (Figure 10) [79]. A radioisotope generator is also continuing to power *Voyager 2* almost 45 years after launch. The most current model of RTG is claimed by the Department of Energy to provide about 110 W of electrical power upon initial deployment while there are conflicting claims about the system on *Cassini* with the DRA claiming the system used for *Cassini* was already capable of 1 kWe and the department of energy claiming 300 We [3,78]. RTGs are generally safe for humans to work around, with the generator designed to survive failed landings and not leak radiation unless it was opened with intention. However, as evidenced by current examples, they lack the power production of other sources. The Mars DRA calls for generators that produce 5 and 10 kWe, with a 5 kWe calculated to measure at 450 kg. At the moment, the technology for these advanced power systems is still in development.

The exact power requirement for our missions is yet to be determined and relies heavily on the state of electrical infrastructure at the time, so the quantity and size of these generators cannot be determined, but that should not stop the development of more efficient RTGs, safer fission systems, and robust solar arrays. There could also be a benefit to over preparing for these missions like the current plan for new aircraft carrier systems. The new Ford-class carriers for the navy are being designed with nuclear power generators to provide enough power to provide capacity for future weapons. The same can be done for Lunar or Martian bases, fly out a large enough generator that new missions and technologies can be adapted to it in the future with existing infrastructure. Further discussion of these power systems will be reserved for other review.

### 5.3. Reactor Design

Multiple photobioreactor designs have been created, though the ideal reactor design has yet to be decided. Huang et al. performed a review of existing proposals for designs that we will reference for this section. There are four main photobioreactor designs that have been considered viable for mass production of microorganisms during spaceflight. The first that we will mention is the plastic bag PBR, a series of plastic bags of culture fixed to metal stands. The major advantage of this design is the low cost of the materials involved and the agitation process. However, the disadvantages of this system are numerous [80]. Gravitational effects distort the shape of the bags, leading to photolimitation of the interior cells in the wider parts of the bag. Plastic is also a frail material, leading to frequent breaks and leaks. Lastly, cleaning has been proven difficult, leading to the need for frequent replacements of the bags over time [80]. The next design is a column-airlift design, a vertical tubular design with a central column in the culture where the input gas is injected, rising to the top as a form of agitation. For that agitation method, this design boasts low operating costs, decreased shear stress on the culture, and good mass transfer. On the other hand, the design does cause difficulty in cleaning and has a higher initial development cost [80].

**Figure 11:**
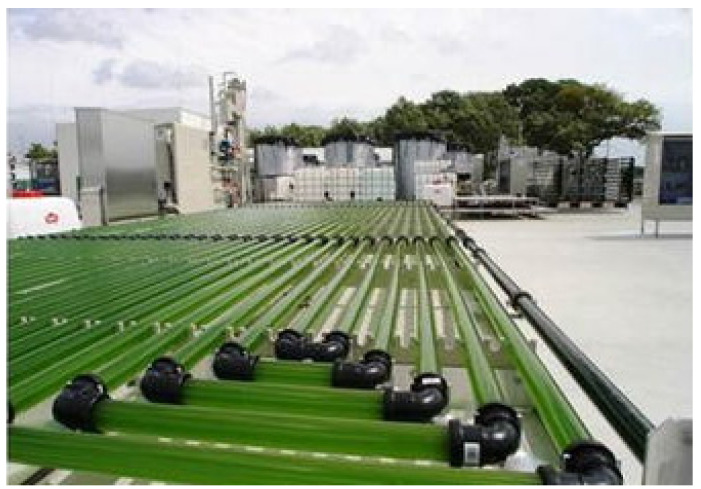
Example of a tubular photobioreactor [81].

**Figure 12:**
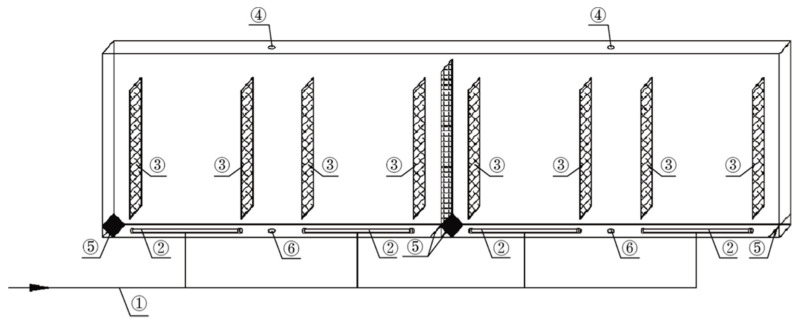
Design of a flat-panel airlift design. (**1**) Air supply tube, (**2**) gas sparger, (**3**) baffle plate, (**4**) air hole, (**5**) dam board, and (**6**) slurry outlet [80].

The third design is the tubular PBR which involves a set of clear tubes, glass or plastic, with a pump used for circulation/agitation (Figure 11) [81]. This design is considered one of the more feasible designs as it is a simpler design associated with a larger relative surface area to volume ratio to maximize light exposure as well as adaptability in shape of the design to fit any space [80]. Photolimitation can be minimized in this design by reducing the diameter of the tube. While one source mentioned most were around 10 cm in diameter, Huang et al. claims that the maximum allowed diameter is 6cm and that diameters of 1cm can produce higher cell densities [72,80]. This design can be plagued by toxic increases in oxygen concentrations when not circulated well, leading to culture death [80]. This is avoided by keeping the length of the tubes shorter. Cleaning this design is currently done by mechanical means. The last type of PBR is flat panel, which is basically a long, thin box that also has a large surface area to volume ratio like the tubular design, leading to a mitigation of the photolimitation [80]. It is easily cleaned and has a long lifespan. Huang et al. patented a design building on that to make a flat panel airlift reactor. This design combines the ease of cleaning and maintenance with the effectiveness and benefits of a column airlift PBR [80]. Their schematic is shown below in Figure 12 [80].

Choosing the final design should involve consideration of both effectiveness and ease of maintaining the reactor. These reactors will have to run autonomously for up to two years before the crew is able to arrive and perform maintenance. Autonomous cleaning systems will have to be put into place to prevent bacterial films from adhering to the surfaces of the reactors and prevent light from reaching the center of the cultures. With that in mind, the plastic bag and column airlift reactors are likely not ideal designs due to the difficulty we have cleaning them here on Earth. Both the tubular and flat panel designs are easy to clean and probably easier to develop automated systems for. Simplicity of the design leans towards the tubular, flat plate, and plastic bag designs, but the over-simplicity of the plastic bag with its failure rate would not be suitable for a mission-critical system so should be eliminated. Tubular and flat plate designs can be made to have interchangeable parts and can be modular for redundancy. Ultimately, these two will be the final two designs for consideration with the current state of development in the field. If they are given adequate lighting and gas exchange, they will each perform well on a mission.

### 5.4. Autonomous Production

Due to the production rate of methane and food stores from BLSS and ISRU reactors, it becomes necessary to align with the DRA 5.0 design to preposition reactors up to 2 years before the arrival of crew. This equipment will have to function on its own to provide food redundancy, as well as methane and oxidizer for the DAV. Adequate storage will preserve the product outside of the reactors and methods to do so will be discussed in the next section. However, what happens when the storage capacities are met and either the crew has not arrived, or the product is not immediately going to be used to clear up storage space? These reactors must have to have a method of pausing the metabolic pathways of the bacteria inside of them; otherwise, the product will build up inside of the reactors, forcing either conditions that are poisonous to the cultures in the case of oxygen or necessitating venting of the product into Martian and Lunar space, possibly contaminating the environment and wasting resources.

The most likely way to accomplish this will be to lower the temperature of the culture to invoke a dormant state. At 17 °C, *Spirulina* enters a dormant state where biological processes stop, but viability continues [71]. A study looking at the long-term storage of *Chlorella vulgaris*, *Euglena gracilis*, and three other microorganisms found that when stored in temperatures between 9 and 20 °C and experiencing reduced illumination at 50–100 lux, cultures of these cyanobacteria remained viable for 12 months or more [82]. One point of difficulty that this experiment had was due to the pH environment of *Euglena gracilis* culture. Like our previous section mentioned, *Euglena* grows in a pH environment more neutral than other species of interest, leading to increased rates of contamination, which is exactly what this experiment experienced [82]. Contamination notwithstanding, when these dormant cultures were placed back into fresh medium after dormancy, their normal growth rates resumed.

### 5.5. Prepositioning of Assets

The timing and contents of pre-positioned elements for a Mars mission varies based on the mission type. Current plans for short term missions only suggest prepositioning the DAV due to the crew only being on the planet surface for a maximum of 30 days whereas long-term mission designs suggest the need to pre-position more of the supplies, including a surface habitat (SHAB). Due to orbital positions and payload mass considerations, pre-positioned items would have to launch separately, about two years before the crew [3].

Among the established benefits of pre-deployment is reduced mass of the primary vehicle stack, allowing for cheaper, more efficient propulsion for the transit vehicle. Trajectories for these pre-deployment unmanned missions can be set on minimum-energy trajectories, conserving fuel and financial resources. Prepositioned ISRU elements can autonomously begin to produce the consumable materials needed for the mission. Staggering launch windows would allow the capabilities of the current facilities at KSC to keep up with the expected increase in demand without necessitating drastic infrastructure changes. Further, if mission elements are sent to the Moon and Mars prior to astronaut arrival, constant communication with NASA would allow for verification of functionality before the crew are in a situation that depends on these systems [3].

Prepositioning a PBR alongside the fuel reactor would also require proper means of storage for the product until the mission crew arrives. The main avenues of storage for cyanobacteria are frozen, freeze dried, and oven-dried, all of which were reviewed by Papalia et al. In their findings, each method displayed different levels of availability of key proteins, antioxidants, and pigments. Comparing the presence of phenolic compounds to fresh *Arthrospira platensis*, oven-dried biomass lacked quercetin and caffeic acid, frozen lacked *p*-cumaric and ferulic acids, and freeze-dried biomass lacked catechin, caffeic acid, and *p*-hydroxybenzoic acid (Table 2 and Table 3) [83]. On the other hand, membrane degradation during the storage process does allow for increased availability of biomolecules contained in the organism. If approached from a numerical perspective, the frozen and the oven-dried methods provide the most preservation of natural benefits. However, both require large amounts of power to dehydrate the product, and in the case of frozen biomass, large amounts of power for continual storage. For this reason, the trade-off of reduced quantities of phenolic compounds (among other biomolecules) for easier long-term storage might have to be made by choosing the freeze-drying method. Once preserved, freeze dried biomass does not require a long-term power source for storage and instead can sit in stasis until the crew arrives on the planet. In fact, *Nostoc muscorum* was successfully lyophilized to test this concept, and it was found to remain viable after five years [84]. Retained viability of the cultures during the process leaves the door open to having multiple backup cultures in case the main production culture dies.

Another reason to go with lyophilization versus freezing, is that it protects against the risk of mechanical failure. If a freezer were to fail, then its entire contents would be wasted. This waste could potentially be mitigated by the cold temperatures experienced in some shaded areas of Mars but as evidenced by temperature data from the Spirit rover, shaded regions can still reach upwards of 35 °C [61]. Again, comparing freeze-dried storage versus oven-dried, de Faria Neves et al. showed that when directly compared to hot air drying, freeze drying is the superior method for maintaining nutritional quality of the biomass, including higher retention of proteins and sugars [85]. Hot-air drying also causes structural damages to the cyanobacteria, leading to absence of viability for recolonization [85].

A drawback to consider for prepositioning elements of the mission is the long deployment time before the crew arrives. Martian weather and dust storms can become formidable events with winds reaching upwards of 70 miles per h [63]. The weather should be taken into consideration when building prepositioned elements so that they can retain integrity and function for not only the time required by crew on the ground but also the time that they are needed to survive on their own. Constant communication is needed between these elements and NASA for verification of functionality. For that, a reliable source of energy is needed.

### 5.6. Redundancy

Redundancy is always a struggle for spaceflight missions due to the constraints on what can be packed for the mission. Long discussions are held to determine acceptable risks and available mitigations. Biological failure, or culture death, is a real danger when relying on living organisms for consumables. This failure was exhibited in the recently released Netflix film *Stowaway* where the crew aboard the transit vehicle needed an alternative method of carbon dioxide scrubbing and a source of oxygen after some of their systems were damaged. They had a dormant cyanobacterial culture on board intended for use on Mars but were forced to use it for transit vehicle atmospheric revitalization. Unfortunately, all the culture died soon after implementation due to equipment and conditions, and the crew were left with limited options. Though this example did not provide the fictional crew members with the equipment needed to handle a culture, it does highlight the need for backup cultures for redundant systems, for atmospheric revitalization, food, and biofuel production. Storing preserved cultures for future repopulation of reactors is essential for these emergencies. *Spirulina’s* growth limitation at colder temperatures is a useful quality for storage as 17 °C should not be hard to maintain from a power perspective. Freeze-drying some of the biomass would also provide an avenue towards redundancy as the process does allow for reuse of those cultures [85].

Redundancy in the equipment can be considerably more difficult than redundancy in the cultures. While seeding samples for cultures are easily regrown and stored from viable reactors, redundancies in some equipment do not have the same luxury. Some smaller pieces may be able to be 3D-printed for replacement but others, like glass tubing, may have to be flown to the site. It will be important to include as many BLSS/ISRU components as possible into a digital printing library, to fit the redundancies into the mass and volume limits of the mission. As for the components that cannot be printed, designing a simple modular reactor with a shared list of parts able to be used in multiple places will benefit the mission. Using the same glass tubing pieces or the same screws and harnesses for the entire reactor will ensure that the list of spare parts is minimally sized and more efficiently packed. Modularity itself would provide redundancy in cases of both mechanical failure of the process or biological failure of culture death because if one culture fails, then many more will still be functional, and production is minimally compromised [72]. As far as computers, temperature controllers, and other electronics go, these will have to be analyzed for potential failure rates and replacement components of the most failure prone components must be packed, since we do not currently have electronics manufacturing capability on the Moon and Mars. For light sources, current digital printing capabilities do not include printing light bulbs, LEDs, or fiber optic cables as would be used for a mission. Thus, all of these would have to be transported to the destination for backup. The exact number of redundant parts will be determined by the lifespan of the light source chosen for the reactor. As an example, with the longest lifespan, LEDs last about 50,000 h. If they were run continuously, that would be about 5.7 years. However, theoretically, they should last for a longer time period given that the reactors may require periods of darkness to function properly.

**Figure 13:**
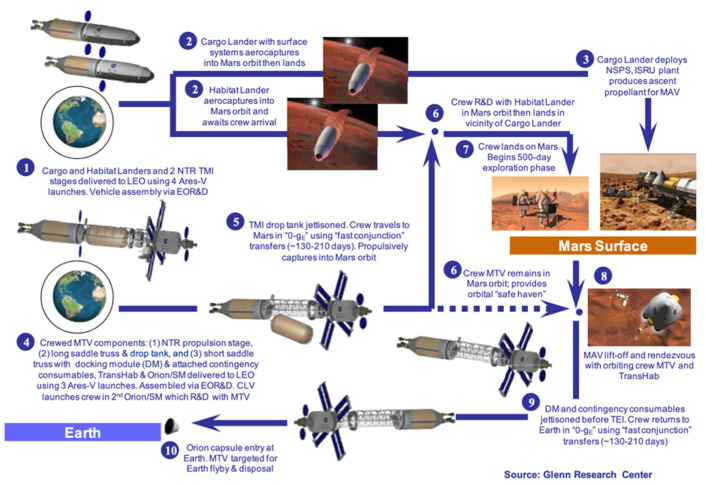
DRA 5.0 long-stay mission overview [3].

Since BLSS/ISRU systems are still in their infancy, physiochemical redundancies should be packed as well. For the planetary ISRU system, this will likely apply more to conjunction-class missions. Opposition-class missions will have the luxury of not relying on ISRU systems on the ground for too long (30-day stay on the surface) and with Mission Control monitoring the ground systems before crew arrival, they can tell whether the resources have been produced prior to landing. If the ISRU system fails and not enough fuel was created, then the mission does not have to land, and the crew can slingshot around the planet to come home. If the production system fails when the crew is on the ground on an opposition-class mission, then the fuel created for the DAV will have already been made before landing was authorized, so crew life is theoretically not endangered. Conjunction-class missions, on the other hand, cannot always abort early due to the orbital positions of the planets, so more resources will have to be stored on-hand. A couple of strategies exist to account for failure. A similar approach could be taken like that of the opposition class and wait for enough resources to be built up for the long stay on the ground before Mission Control gives the order to land. This will undoubtedly take a longer prepositioning window, but if the production and storage infrastructure can survive, then it is possible. An alternative strategy is to fly out reserves of the required materials with the crew to ensure human survivability for either the duration of the ground mission or until an emergency resupply mission could travel there. This would require multi-ton payloads including up to 40 T of fuel and many more of food, oxygen, and carbon dioxide scrubbers. The cost of covering disasters leads to fewer scientific experiments and less overall capability. An example of an extended-stay missin that incorporates these principles can be found in Figure 13 [3].

The ideal preparedness scenario for a conjunction-class mission would probably be a combination between the two strategies discussed above. Prepositioning the reactors and ensuring the resources exist would be the safest plan, as you can guarantee that they exist, but in case the storage facilities fail, we should prepare for shortages based on how long it would take for a resupply probe to reach the planet. The farthest approach to Mars is 289 days with the average being only 162 days not before considering the time to create the probe, mount it on a booster, test it, and launch [86]. The safest option would be to plan for about 300 days of backup supply, while the NASA team on Earth has a fast-tracked probe creation strategy on deck for efficiency. Anything beyond that, and it would be more worthwhile to send supply for the whole mission from the start. Of course, all of this will become less necessary as we build and demonstrate more robust ISRU systems that can survive the harsh conditions of Mars.

### 5.7. Scalability for Crew

In the previous section describing the BLSS application for food for the crew, we discussed the calculated reactor size needed to support a 3–4-person crew. We calculated the need for 17,250 L of reactor before considering redundancies and the need to harvest some biomass for the biofuel stage of ISRU. However, just as has happened for the ISS in the shuttle era, what would happen if we had a crew turnover shift with an increase of crew size to 8? Assuming a turnover duration of 30 days, approximately the lower bound duration of stay for an opposition-class mission, we would need enough food for the turnover crew for that time.

A few designs can take place here. First, if the existing reactor has been producing just enough for daily harvests of food for the crew and was producing and storing biomass and oxygen for the two-year period before the outgoing mission, then there should be plenty of reserves to tap into to provide nourishment and air for the turnover crew for 30 days barring any complication with the reactor. Second, multiple reactors could be established for support. If a different DAV is used for each mission much like was used in the novel *The Martian*, then it would be reasonable to assume that this could be deployed with many of the prepositioned elements of previous missions, including more reactors. This would also add an extra layer of redundancy for the mission. The extra reactors would then be in production mode before the turnover crew arrives, building up stores and capability for that crew to use.

Alternatively, another option is to just pack the 30 days of food, oxygen, and carbon dioxide scrubbers with the incoming mission. This option would allow a reduction in the mass shipped to Mars by eliminating the need to ship an extra reactor and relying on the existing reactor from the outgoing mission, provided it is still within its service life. Cost and material efficiency would peak in this scenario, though analysis on whether the existing reactor would survive another conjunction class duration mission would be required. The final scenario would be to increase the production rate of the existing reactor to meet the needs of the new crew. With the established maximum growth rate for *Spirulina* at 87% daily growth, using a “ramp-up” plan from a lower basal efficiency to a higher one to get extra production would require the reactor to operate at sub-peak efficiency for the duration of the outgoing mission, and still provide the daily requirements for the outgoing crew for that duration. Operating at 50% peak efficiency would require twice the volume of reactor space, meaning the same number of resources would be used as in deploying multiple reactor units for each mission as discussed before. The “ramp-up” plan would only be cost-effective if the larger reactor was used over the course of three or more missions, provided again that they do not have a service life below that.

## 6. Inflight BLSS

BLSS systems in place for transit, some could argue, are the most taxing to the mission because this reactor cannot be prepositioned or flown in a separate vehicle like the planetary ISRU system. Instead, it must produce its contents from launch to landing. For our discussion, we are going to assume that this system will be providing only atmospheric revitalization and biomass for human consumption, not fuel production, because all fuel for the transit vehicle is being provided from launch for the whole mission.

### 6.1. Nutrition

The composition of the U.S. baseline diet generally acceptable for space flight, based on considerations from the International Space Station, consists of about 50% of the kcal coming from carbohydrates, 17% from protein, and 31% from fat. These nutritional requirements have been designed for astronauts who usually engage in moderate levels of daily activity. It has been realized that for a BLSS designed for long duration space flight, food supplied in space must not only serve nourishment needs but must also contain biologically active components that deliver benefits beyond basic nutrition, such as health promotion, prevention of disease, and psychological health [87,88]. In this section, we will discuss supplying pre-packaged foods as well as bioregenerative food systems for long duration missions without repeating the selection, details, and benefits of eating cyanobacteria and microalgae as it was covered in the Planetary ISRU section.

Pre-packaged foods such as dried meats and other assorted foods could help meet some of the nutritional requirements according to NASA. Assorted spices, bouillon, sugar ingredients, dried egg ingredients, water, starch, and assorted dried pastas can constitute the resupplied ingredients [89]. With regards to ready-to-eat crops that are grown in situ, cabbage, carrots, celery, green bell pepper, beans, rice, spinach, and strawberry fit this category [90]. However, these foods take a long time to grow. Some of the advantages of a bioregenerative food system that is part of a BLSS are sustainability, recycling of gases and waste products, and flexibility to customize dishes according to personal preference of crew members since the basic ingredients are directly grown while in flight. Importantly, the quantity of shipped mass is greatly reduced with food that can be grown in space leaving room for other necessities. A bioregenerative food system also largely provides for a diet that is organic in nature, which suggests some potential benefits for long-term health [90]. One disadvantage is the greater amount of time crewmembers will dedicate towards agriculture and food preparation tasks. Another disadvantage, particularly while in flight, is that there is limited protection from damaging radiation of solar flares as the spacecraft will spend great amounts of time outside of the vicinity of any planetary magnetic field. Cultivated organisms in a BLSS can be damaged by such dangerous levels of solar radiation and protective technology for this continues to be developed. Moreover, reduced gravity and microgravity affect water and nutrient delivery for root functions and higher plant growth as well as diffusion of gases and waste. Hence, specialized equipment, such as an irrigation and nutrient delivery system which are effective in a microgravity environment is needed [91].

Cyanobacteria and other microorganisms can provide the solution for these missions, especially with the nitrogen starvation of said microorganisms leading to higher carbohydrate content. One challenge for running a bioreactor during transit is the lack of regolith to supply the nutrients. The reactors will need input as well which will have to come from either a pre-packed tank of solution (probably Zarrouk’s) or, in the case of a return trip, pre-packed regolith from the Martian or Lunar surface. Elemental recycling systems like human waste and air scrubbers could close the ecosystem, but inefficiencies will only lead to a loss from the system. Since conservation is important, if Zarrouk’s medium was used, it has been shown that diluting it up to 50% has shown no loss in productivity [52].

### 6.2. O_2_ Production

As discussed previously, oxygen production in current spaceflight missions is provided through various physiochemical means like solid oxygen releasers, electrolysis, and byproducts from fuel consumption during launch. As stated in the Planetary ISRU section, oxygen evolution has been marked at 227.2 g/kg biomass for a cyanobacterial PBR with the size of the PBR needing to be at least 3918 L to support a four-person crew when only exposed to an 8 h lighting period. The size of this reactor should increase with increased EVA and IVA activity for the crew to account for increased metabolic rate. The produced oxygen could be used for life support and generating electrical power via fuel cells and propulsion of the spacecraft. However, any additional O_2_ that is produced but not immediately utilized must be purified and stored efficiently. Solid state electrochemical oxygen separation and compression currently provides an efficient means for achieving >99.99% O_2_ purity [92,93]. Requiring a total of 470 W of power, this method operates at 2 L/min and forms pressurized oxygen at 200 psi. Oxygen can subsequently be stored in tanks for future use. Alternatively, since oxygen can be used to generate electrical power using a fuel cell, the overall balance of oxygen utilization can be fine-tuned and optimized. Specifically, with a regenerative fuel cell system, using a combination of a primary fuel cell and an electrolysis system, one could generate electricity and/or oxygen from water via hydrolysis [94]. Hence, this system in conjunction with a bioreactor can be used to efficiently meet the in-flight oxygen requirements and generate additional electrical power that could be immediately utilized or stored in a battery.

### 6.3. Carbon Dioxide Scrubbing

The utilization of carbon dioxide (CO_2_) must be analyzed differently from that of oxygen as both high and low levels of CO_2_ have implications for bioreactors and the crew. For an algal photobioreactor, the CO_2_ consumption has been observed at 5.28 g/L-day. Algal (e.g., *Chlorella*) photobioreactor carbon fixation efficiency is currently being improved by generating various mutants using gamma irradiation and optimizing growth conditions such as nitrogen, phosphorus, and magnesium levels [95,96]. A cyanobacterial photobioreactor consumes less CO_2_ on average at about 1.45 g/L-day. Carbon uptake within cyanobacteria is also being improved by modifying bioreactor growth conditions and through genetic engineering of the Ribulose-1,5-bisphoshonate (RuBisCo) enzyme that helps increase carbon fixation within the cyanobacteria [97].

It is important that carbon dioxide levels are carefully regulated in the confined environment of a spacecraft, as confined spaces offer the greatest risk for adverse effects from the displacement of O_2_ by CO_2_. For the crew members, elevated carbon dioxide levels have been associated with changes in brain connectivity, sensorimotor performance, and behavior among other physiological effects [98]. Moderate levels of CO_2_ from 8–10% in the air are sufficient for crew members to show signs of asphyxiation and loss of consciousness. Increasing concentrations of CO_2_ can be rapidly fatal [99]. For a bioreactor, CO_2_ levels that exceed the microorganism CO_2_ consumption rate lead to the excess carbon dioxide acting as a disturbing agent that alters the pH of the environment, with pH being one of the sensitive parameters for efficient functioning of a bioreactor system [100].

Low level of CO_2_ exposure to crew members may not be immediately life threatening but may have health consequences in the form of subtle, seemingly benign effects. Slight increases in CO_2_ tend to increase cerebral blood flow and blood pressure, which can lead to increased intracranial pressure. Prolonged low level CO_2_ exposure leads to increases in lung dead space volume and inefficiencies in gas exchange [99]. For a bioreactor, low levels of CO_2_ simply lead to inefficiencies in carbon fixation as maximum performance for the enzymatic reactions will not be achieved [100].

Optimal CO_2_ concentration for maximizing CO_2_ fixation within a bioreactor seems to likely be a moderate concentration that is neither too high nor too low. Nevertheless, to account for fluctuations in CO_2_ levels and regulate the efficiency of CO_2_ consumption from a physical perspective, the size at which CO_2_ bubbles are introduced into the bioreactor system has recently been found to play an important role [100]. Cultivation of *Spirulina* conducted under varying levels of carbon dioxide showed that growth rate hits a maximum at 0.07% CO_2_ [101] while another study tested ranges from 0.36% to 10% CO_2_ and found that 6% carbon dioxide yielded the highest levels of biomass [102]. The atmosphere of current space vehicles matches with this range as these organisms have adapted to survive in the same atmosphere we breathe on Earth.

### 6.4. Radiation Protection

A particularly important consideration regarding the use of cyanobacteria in regenerative life support systems operating outside of Earth’s protective magnetosphere is the ability of said cyanobacteria to survive exposure to radiation, which is expected on any long-duration mission beyond LEO. Research related to the radio-susceptibility of cyanobacterium species is complicated by the fact that ionizing radiation (IR) in space is complex, temporal, and comes in many wavelengths and particle sizes [103]. As such, it is difficult to adequately simulate the conditions present in deep space on Earth. Recent measurements on the Lunar surface taken by Chang’E 4′s Lunar Lander Neutrons and Dosimetry (LND) experiment indicate an average total absorbed dose rate in silicon of approximately 13.2 mGy/h [104]. The Mars Science Laboratory—Radiation Assessment Detector (MSL-RAD) aboard the Curiosity Rover estimated a total dose equivalent, from both galactic cosmic rays (GCR) and solar energetic particles (SEP), of 466 ± 84 mSv for its 253-day transit between Earth and Mars [105]. Surface experiments at the Gale Crater site indicate Martian GCS dose rates varying between 180 and 225 mGy/d, with a single SEP event occurring during the first ~300 Martian sols accounting for an additional 50 mGy [106]. Based on these measurements, a total Mars mission dose equivalent of ~1.01 Sv is estimated for a reference mission with 180 days of transit time (each way) and a 500-day mission duration [106].

The effects of the aforementioned radiation exposure on the health of a human crew are certainly a paramount consideration and range from acute destruction of sensitive tissues including skin, bone marrow, and intestinal lining to long-term cancer risks from DNA damage. However, it is also important to consider the effects of this radiation on regenerable life support systems, which are biologically active and are therefore susceptible to damage caused by IR. As discussed, cyanobacteria are excellent candidates for use in a BLSS due to their efficiency, nutritional value, and air revitalizing capabilities. From a crew radiation mitigation perspective, some cyanobacteria such as those from the genus *Arthrospira* offer the unique benefit of producing phycocyanin and its chromophore phycocyanobilin (PCB). These molecules act as potent antioxidants which inhibit peroxynitrite (ONOO–) mediated oxidative damage in DNA [107]. By scavenging several biologically destructive free radical species, these compounds, when ingested, may serve to protect astronauts’ cells against damage and oxidative stress caused by IR [108].

*Arthrospira* species have demonstrated considerable resilience towards harsh radiation environments. *Arthrospira* sp. PCC 8005 demonstrated the ability to regrow normally when exposed to a point radiation source up to 1600 Gy (dose rate 526 Gy/h). Further doses of radiation up to 6400 Gy were tolerated, although a delay in regrowth was noted [108]. While there was a temporal impairment in photosynthetic activity following exposure to radiation, *Arthrospira* sp. PCC 8005 was able to recover, proliferate, and resume photosynthetic activity even after exposure to radiation far beyond what is to be expected on Lunar or Martian missions. These findings are consistent with other studies that demonstrate similar resilience amongst many cyanobacteria species to a multitude of spectra of IR, including the UV-C flux present on the Martian surface and X-rays [109,110]. Indeed, it is postulated that the radiation resistance exhibited by cyanobacteria comes from defense mechanisms gained during the Precambrian era, which was marked by an incomplete atmosphere and increased surface IR on Earth [108].

The Biology and Mars Experiment (BIOMEX), housed in the EXPOSE-R2 external exposure facility on the ISS, tested the durability of cyanobacteria exposed to the radiative and desiccating environment of space. In this experiment, the bacteria were cultured on Lunar and Martian regolith analogs and exposed to space, as well as simulated Mars-like conditions. After exposure to 0.5 Gy of IR, as well as the vacuum of space and (in some cases) UV radiation, the samples were inoculated into growth medium for viability assessment. The non-UV exposed, as well as the Lunar and Martian regolith analog samples of *Chroococcidiopsis* sp. CCMEE 029 proved to be viable after 672 of exposure to space in LEO. The UV-exposed, non-protected by regolith sample of *Chroococcidiopsis* sp. CCMEE 029 did not demonstrate viability [111]. Indeed, cyanobacterium Chroococcidiopsis has been known to survive up to 11.59 kGy of gamma radiation and up to 2 kGy of Fe ionic radiation, far beyond what would be expected on any Lunar or Martian mission [112]. Based on the results described above, it is therefore expected that many species of cyanobacteria may prove to be suitable candidates for use in bioreactors and BLSS due to their extreme resilience towards the harsh radiation environment of space.

### 6.5. Redundancies and Safety

As with any system, it is important to keep redundancies in play to ensure safety of the crew and viability of the mission. For inflight atmospheric emergencies (oxygen/carbon dioxide imbalances), relying on a BLSS reactor for quick fixes is likely not to be fruitful. Like any living organism, gas exchange takes time. Ramping up oxygen production and carbon dioxide scrubbing is not a flip of a switch or an immediate change of the reactor environment. Instead, we must wait for new biomass to grow. If the carbon dioxide concentration becomes too high, then its natural effects to lower the pH of aqueous solutions might create an imbalance in the culture, lowering efficiency and making the situation worse. For this reason, it is important to pack some of the physiochemical systems on board as well, especially in the infancy of BLSS systems. We do not currently have an accepted design of what the inflight BLSS reactor will look like, but when it is complete, risk analysis must be done to determine how many spare sources and sinks have to be packed to account for expected failures.

The same should apply to foodstuffs for the transit portion of the mission. If a reactor were to fail during flight, we would want to have a backup for crew survival. The current packaged food system used on the ISS should be improved and expanded to provide meals for the crew while a BLSS system is being installed. If volume and mass are still a problem, then solutions include reducing daily portions allowed in an emergency, increasing the density of the food developed, providing for inflight resupply, or removing the BLSS module altogether. Over-packing within the allowance of the vehicle might have a place in the context of this design.

## 7. Planetary Protection

Any mission to Mars or another celestial body must ensure that the proper planetary protection measures are in place to reduce forward and backward contamination. The importance of planetary protections cannot be overstated. If proper protocols are not in place and followed by expeditions, from governments, private companies, or partnerships, then scientifically relevant sites could be irreparably exposed. Alternatively, if there is a form of life on a celestial body that is transported back to Earth, it could become an invasive species, possibly finding Earth conditions more hospitable than its native environment. As manned explorations of celestial bodies are designed, such exposures must be avoided or planned for with strict safety protocols.

Planetary protection guidelines, as followed by NASA, must comply with the Committee on Space Research (COSPAR), a consultative to the United Nations Committee on the Peaceful Uses of Outer Space. COSPAR designates five categories of protection, depending on the destination’s likelihood to provide “understanding the process of chemical evolution or the origin of life” and the likelihood of contamination confounding future scientific studies (Table 4). Category V has two sub-categories: restricted and unrestricted, depending on the likelihood the celestial body contains indigenous life forms or is of scientific interest.

These rules are to prevent forward contamination to the mission destination and backward contamination into the Earth biosphere. Current NASA guidelines found in the Mars Design Reference Architecture 5.0 designate certain areas as Mars Special Regions. These are areas defined by COSPAR as areas likely for Earth-based organisms to propagate or possibly hold native Martian life forms [3]. Selection is based on water activity and temperatures warm enough to hold earth-based lifeforms (≥25 °C).

For a Mars mission, much of the travel to these regions would require expeditions of rovers recovering regolith for further analysis at a base camp. Such rovers have different allowable levels of bioburden depending on the nature of mission and intended site, with the highest restrictions limiting the bioburden to <0.03 spores/m^2^ [113]. The base camp and manned astronaut voyages would stay within zones designated to be zones of minimum biological risk (ZBRs), which have already been demonstrated to be safe. To prevent forward contamination of Mars, these areas will be cleared by robotic exploration. Any return of such samples to Earth will be under contamination and isolation protocols to prevent backward contamination.

Turning our attention to the nutritional and atmospheric needs of a manned mission, in which a biological life support system (BLSS) is likely to be utilized, we are primarily concerned by possible forward contamination of Mars or other celestial bodies. Thus, there must be protocols and measures in place to ensure that the contained organisms do not confound future planetary studies. Such a BLSS is likely to use cyanobacteria or some other sort of basic algal life. As stated, cyanobacteria are generally resilient to conditions that other forms of bacteria may not withstand. As such, there must be substantial protocols in place to ensure that they do not contaminate the Martian soil. The first consideration will be the design of the bioreactor within which they will be contained and utilized by the astronauts.

### 7.1. Airlock and Containment Measures

Whichever design of bioreactor(s) is utilized, it is almost certain that astronauts will have to go into the bioreactor housing for maintenance or collection of materials. However, this must be done with care to not contaminate the biological system and for astronauts to not inadvertently collect biological material on themselves and seed them onto the Martian surface. This section will discuss technologies utilized today for avoiding contamination of materials and reducing exposure to biologically sensitive materials and how they may apply to the maintenance of a long-term BLSS by a crew.

### 7.2. Biosafety Lab-Like Designs

A comparable situation, in terms of biosafety procedures, on Earth might be found in biosafety labs (BSL). There are a range of BSL ratings from 1–4, where 1 is the least stringent and 4 is tightly regulated, rare labs that are generally under the control of the government and are in isolated and restricted buildings [114]. The protocols of ingress and egress of a BSL-4 are numerous, comprising of a buffer corridor, a change room in which personnel doff the clothes they came in with, a separate room in which the suits are maintained, a chemical shower room that must be passed through before entering the lab, and a shower after doffing the lab suit when exiting.

**Figure 14:**
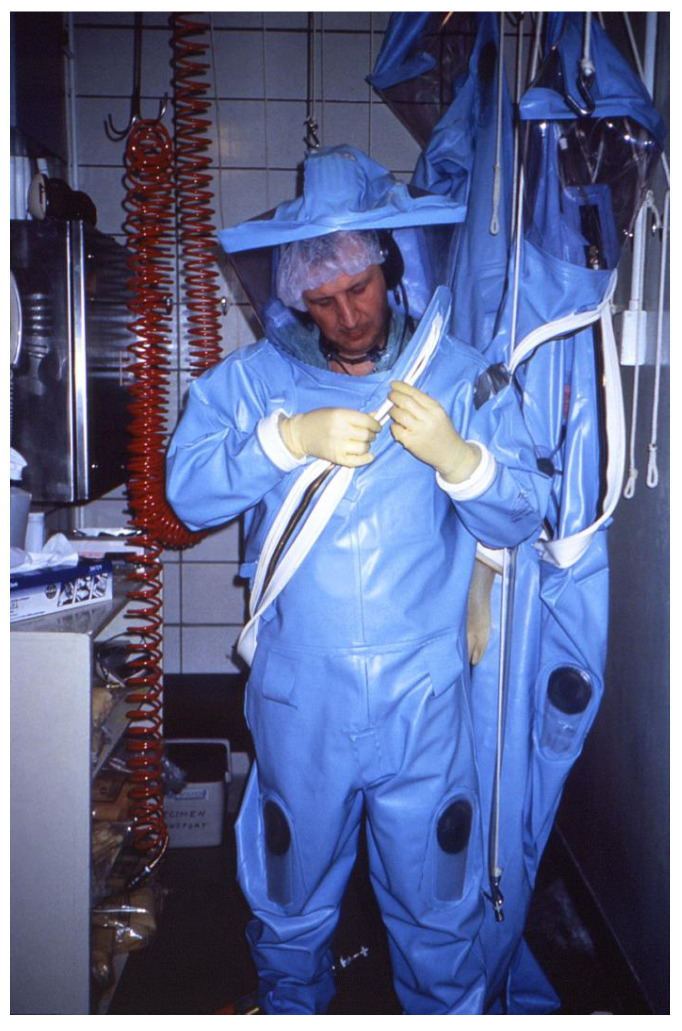
CDC Personnel donning a positive pressure suit [115].

Personnel enter through the buffer corridor, record their visit into a logbook, then enter the change room and disrobe before changing into scrubs. The personnel member can then move into the outer change room to don the suit. The suit is examined for structural integrity and inflated to maintain a positive pressure to prevent any exposure to the hazardous organisms within the lab. When suiting, personnel must don double-glove over an inner, duct-tape secured glove as well as securing the ankles of the suit and any zippers or minor defects with duct tape. Air is supplied directly to the suit via a tube to maintain circulation for the personnel member and to maintain positive pressure within the suit. The personnel member will then request access to the chemical shower room that is secured by doors with inflatable bladders that the personnel member must wait to deflate before passing through, creating a form of airlock between the change room and the lab. An example suit is shown below in Figure 14 [115].

When doffing, the personnel member goes through the chemical shower room to clean the suit they are wearing and afterwards will inspect it for any flaws. The chemical showers use many detergents, among which is Micro-Chem Plus, a dual quaternary disinfectant that is advertised to have bactericidal properties against many disease-causing bacteria, virucidal, and fungicidal properties and against trichophyton mentagrophytes when used on non-porous surfaces [114]. While there is not a wealth of information on the efficacy of dual quaternary disinfectants to kill cyanobacteria, alkyltrimethylammonium surfactants show promise as inhibitors of photosynthesis of cyanobacteria [116]. The gloves are examined to ensure the outer gloves are intact. Finally, the personnel member will shower on-site, place their original clothes on, and leave [114].

This has the potential to be adapted to a biosafety lab-like room for the bioreactor. The astronaut in charge of maintaining the bioreactor would follow a similar protocol. However, there are certain adjustments that would have to be made for adapting this protocol to a Mars environment. For example, bringing duct tape used to secure the gloves to the suit, as done on Earth, would create unsustainable waste on Mars. Similarly, sending enough material to Mars to construct separate rooms for showering, changing, and a separate chemical shower room is likely to be impractical.

A likely way that this process could be adapted to Martian use would be the use of designated bioreactor room suits, a separate airlock that crewmembers must pass through to access the bioreactor room, and, of course, the inspection and maintenance of the suits and other associated gear, similar to the inspection of the BSL-4 labs. Current suits used by BSL-4 personnel are made of various polymers with a hydrophobic coating, such as neoprene or PVC [117]. Suits used by the crew members in maintenance and use of the bioreactor could utilize something similar—a hydrophobic material that would reduce bacterial adherence and excess moisture exposure causing suit rot. To tackle this problem and others, Dr. Lawrence Kuznetz and Planetary Protech have developed a “Q-suit” suitable for all IVA’s that has also found a use during the COVID-19 pandemic to stop the spread of infection. Their design also boasts ease of donning and doffing, Bluetooth connectivity, microscopic protection, and full isolation with waste, water, and food management onboard [118]. Future developments like these will be key to providing a clean environment to carry out BLSS/ISRU activities.

### 7.3. Ultraviolet Decontamination

Another possible technology that could be adapted to prevent contamination of the main habitat from the bioreactor is ultraviolet decontamination systems. Currently ultraviolet systems are used in many decontamination systems such as biosafety cabinets, air filtration systems, hospital disinfection, etc. In studies investigating the efficacy, UV has been shown to have a high capacity to kill microbes found on humans with elevated times of exposure needed to inactivate particularly resilient organisms, such as anthrax spores, *C. difficile* and *P. aeruginosa* biofilms [119,120,121]. There is not a wealth of knowledge on the ability of ultraviolet to kill cyanobacteria; however, ultraviolet-A (UV-A) can cause negative effects on cell concentration, and UV-B, while not disrupting cellular concentration, can reduced cell viability in cyanobacteria [122,123]. The ability of cyanobacteria to be aerosolized is well documented in several regions and is dependent on atmospheric conditions such as temperature and humidity [124,125]. It may be a useful design to select a species for the bioreactor that does have sensitivity to UV decontamination systems. Additionally, there could be ultraviolet decontamination systems utilized in the suit room to prevent microorganisms from the crewmembers from infecting the bioreactor colony and compromising its efficacy.

### 7.4. Current Regolith Handling

Other isolation techniques to draw from are NASA’s own regolith sites such as the Lunar regolith storage at NASA. The Lunar regolith at these sites is stored in special airtight containers which are purged with pure nitrogen. The containers have their own three-layered gloves embedded in the wall that can be used to handle the regolith. The only tools that are allowed to contact the samples are packaged in hermetically sealed bags and are made of aluminum, stainless steel, or Teflon [126].

## 8. Substrate Addition and Harvesting of Bioreactor Products

The purpose of the bioreactor is to serve as BLSS for the crew. As such, there will be material exchanged between the BLSS and the main habitat environment in the form of gasses and nutritional biomass. Safely moving these materials between the two without transmittance of biological specimens to the Martian surface is of the utmost importance.

### Substrate Addition Challenges

Having a simple connected air system would defeat the purpose of all the above-mentioned isolation techniques. However, carbon dioxide must be fed into the system, and oxygen must be collected from the bioreactor to maintain the atmosphere of the main habitat. Again, we can look to preexisting filtration and isolation technology to incorporate in this design. Already mentioned above is the use of ultraviolet decontamination systems in air systems. Such a design may prevent contamination from the main habitat to the cyanobacteria; however, it may do little to stop the spread of cyanobacteria to the main habitat. A design that may be of more use would be an air scrubber system and an oxygen concentrator, such as a HEPA filter utilized in an isolation system [114]. Similarly, commercial oxygen concentrators are available with sieve beds that last roughly 12–18 months [127]. A greater challenge would be a carbon dioxide concentrator for injecting carbon dioxide into the cyanobacteria reactor. Currently, concentrating carbon dioxide from a mixed gaseous atmosphere is possible, and systems to do so are utilized in the NASA spacesuit design. However, the release of the carbon dioxide from the system is based on a favorable gradient. As such, most carbon dioxide scrubbers are simply waste products taken back to Earth; however, newer, more promising systems are under investigation [3]. Such concentrator systems will require advances in design in order to become feasible for sending one on an extended trip to Mars where there will be limited repair possibilities.

Another design challenge is the addition of regolith and other substrates to the bioreactor. Every activity done within the director room is a possible source of forward and reverse contamination. Thus, the possibility of utilizing a robotic system or a control system for the addition of regolith and other substrates to the bioreactor should be fully explored. Another possibility would be the addition of regolith directly through a port that opens to the Martian surroundings. However, again this increases the likelihood of contamination. If regolith and other materials are to be added in the bioreactor room by humans, they should follow all the protocols deemed necessary for such hazards. Alternatively, there should be a technology that allows for addition of substrate with minimal chance of contamination of the bioreactor. Such possibilities include a type of adapted biosafety cabinet with a difference in pressure from the surrounding area [128].

## 9. Conclusions

BLSS/ISRU systems will be a key component of any long-duration space mission in the future, a continuation of the effort to make exploration spaceflight more feasible and affordable. Current physiochemical systems are effective in their implementation but do not provide the sustainable reusability that a long-duration mission would require. ISRU-based regolith processing with physiochemical systems would require either high temperatures associated untenable power draws or with highly caustic and non-replenishable reagents. A well-designed BLSS system for both ARV and ISRU can potentially allow the needed renewable sustainability without the major drawbacks of regolith processing. A cyanobacterial-driven photosynthetic reactor may be the optimal choice to provide this reusability. When properly scaled and grown in the right conditions, cyanobacterial bioreactors can produce enough resources to support a four-person crew. Additional bioreactor modules, with proper storage mechanisms and defined enhancement procedures, can allow production to be scaled up for an increased crew size.

Though many bioreactor photosynthetic species have high concentrations of proteins, work has been done to show that nitrogen starvation yields higher concentrations of carbohydrates, thereby molding the organism’s composition to be closer to human dietary needs. Oxygen production is effective and can be scaled up to include production of excess liquid oxygen for use as the oxidizer for the fuel of the descent/ascent vehicle. When looking for an ISRU-based return fuel, good fortune favors the additional property of cyanobacteria as a potential methane producer, a rocket-fuel with notably high I_sp_. While production of biomethane still needs to be refined, vehicle engineering would also need to be modified based on ISRU capabilities, as there has not been widespread adoption of LOX/methane engines in the Lunar/Mars DRM to date.

Finally, bringing another new life form, in addition to humans, to a planetary location where a primary mission of the humans is to search for evidence of extant or extinct life makes it imperative to protect that planet from forward contamination. Sterilization and containment procedures are key to preventing the microorganisms we bring from Earth from creating a new ecosystem on another planetary body. While challenges remain in the field of planetary exploration BLSS/ISRU, as proposed designs still need to be put through rigorous field testing, the future for this evolving technology looks as bright as a Martian sunrise.

## Figures and Tables

**Table 1:** Summary of atmosphere contained in previous spaceflight missions.

Project	Mission Duration	Cabin Volume (M^3^)	Crew Size	Technological Approach
Mercury	34 h	1.56	1	Atmosphere: 100% O_2_ at 34.5 kPa Atmosphere supply: Gas at 51.7 mPa CO_2_ removal: LiOH Trace contaminants: Activated carbon
Gemini	14 days	2.26	2	Atmosphere: 100% O_2_ at 34.5 kPa Atmosphere supply: Supercritical storage at 5.86 MPa CO_2_ removal: LiOH Trace contaminants: Activated carbon
Apollo	14 days	5.9	3	Atmosphere: 100% O_2_ at 34.5 kPa Atmosphere supply: Supercritical storage at 6.2 MPa CO_2_ removal: LiOH Trace contaminants: Activated carbon
Skylab	84 days	361	3	Atmosphere: 72% O_2_/28% N_2_ at 34.5 kPa Atmosphere supply: Gas at 20.7 MPa CO_2_ removal: Type 13X and 5A molecular sieves regenerated by vacuum swing Trace contaminants: Activated carbon
Space Shuttle	14 days	74	7	Atmosphere: 21.7% O_2_/78.3% N_2_ at 101 kPa Atmosphere supply: Gas at 22.8 MPa CO_2_ removal: LiOH Trace contaminants: Activated carbon and ambient temperature CO oxidation
International Space Station	180 days	Up to 600	3 to 6	Atmosphere: 21.7% O_2_/78.3% N_2_ at 101 kPa Atmosphere supply: Gas at 20.7 MPa/water electrolysis CO_2_ removal: Silica gel with type 13X and 5A molecular sieves regenerated by vacuum/temperature swing CO_2_ reduction: Sabatier reactor Trace contaminants: Activated carbon and thermal catalytic oxidation

**Table 2:** Phenolic compounds in *A. platensis samples* (compounds present in the biomass are denoted by (+) those missing are denoted by (−) [83].

Compounds	Rt	*A. Platensis* Fresh	*A. Platensis* Oven-Dried	*A. Platensis* Frozen	*A. Platensis* Freeze-Dried
Gallic acid	6.11	+	+	+	+
Catechin	11.28	+	+	+	−
Caffeic acid	13.22	+	−	+	−
*p*-Hydroxybenzoic acid	14.13	+	+	+	−
*p*-Cumaric acid	18.69	+	+	−	+
Ferulic acid	18.81	+	+	−	+
Quercetin	29.59	+	−	+	+
Genistein	34.95	+	+	+	+
Kaempferol	36.67	+	+	+	+

**Table 3:** Total proteins and antioxidant molecules in *A. platensis* biomass fresh and differently processed [83].

Biomolecules	*A. Platensis* Fresh	*A. Platensis* Frozen	*A. Platensis* Oven-Dried	*A. Platensis* Freeze-Dried
Total Proteins (mg g D.W.^−1^)	188.60 ± 13.55b	283.96 ± 11.79a	122.73 ± 2.53c	167.09 ± 4.35b
Ascorbic acid (mg g D.W.^−1^)	1678.29 ± 2.62b	3149.54 ± 7.99a	354.79 ± 0.93d	1403.9 ± 11.49c
Dehydroascorbic acid (mg g D.W.^−1^)	1998.99 ± 7.01c	1362.93 ± 22.78d	3296.69 ± 15.56b	4660.74 ± 34.56a
Total Phenols (mg g D.W.^−1^)	15.77 ± 1.10b	22.65 ± 0.46a	12.14 ± 1.84c	11.91 ± 0.28c
Total Flavonoids (mg g D.W.^−1^)	20.82 ± 0.08b	8.04 ± 0.29c	4.31 ± 0.11d	30.92 0.17a

**Table 4:** COSPAR Planetary Protection Summary.

Category.		Description	Protections	Possible Destinations
I		Not of interest for origins of life or evolution	None	Asteroids
II		Interest for origins of life and evolution; remote chance of contamination	Planetary Protection Plan, pre- and post- mission reports, documentation	Comets, Pluto
III		Targeted body of interest for origins of life and evolution, significant chance of contamination, flyby or orbiter missions	Cleanroom preparation, additional documentation from Category II	Mars, Europa (Orbiters)
IV		Targeted body of interest for origins of life and evolution, significant chance of contamination, probe or lander missions	Category III requirements, sterilization techniques, bioburden reduction	Mars, Europa (Lander)
V	Unrestricted	Earth-return mission, no indigenous life	Protections determined by fit into above categories	Mars, Europa
	Restricted	Earth-return mission, possible indigenous life of scientific importance	Highest restrictions with containment throughout entire return phase	Venus, Moon
